# Barrier-to-autointegration factor protects against the cGAS-STING response to chromatin bridges

**DOI:** 10.1371/journal.pgen.1012191

**Published:** 2026-06-03

**Authors:** Laura Chastant, Karine Normandin, Firas El-Mortada, Marc J. Servant, Vincent Archambault

**Affiliations:** 1 Institute for Research in Immunology and Cancer, Université de Montréal, Montréal, Québec, Canada; 2 Faculté de Pharmacie, Université de Montréal, Montréal‌‌, Québec, Canada; 3 Département de biochimie et médecine moléculaire, Université de Montréal‌‌, Montréal, Québec, Canada; UMass Chan Medical School: University of Massachusetts Chan Medical School, UNITED STATES OF AMERICA

## Abstract

Cellular damage or stress can lead to disorganization, mislocalization or damage to self-DNA that can activate intracellular innate immune response mechanisms. Micronuclei, such as can occur following mitotic defects, have been proposed as a source of DNA capable of activating the cGAS-STING pathway, resulting in IRF3-dependent proinflammatory transcription. However, to what extent micronuclei *per se* or other concurrent defects contribute to the cGAS-STING response remains unclear. To better understand the ability of post-mitotic defects to induce this response, we compared the effects resulting from inhibition of the Spindle-Assembly Checkpoint (through MPS1 inhibition) or interference with nuclear reassembly (through inactivation of BAF). We found that combining both perturbations synergistically enhances the cGAS-STING response. This effect is not due to an increase in post-mitotic nuclear deformations including micronucleation and lobulation but instead correlates with an increase in destabilized chromatin bridges resulting in structures that potently recruit cGAS. Our results suggest that by stabilizing chromatin bridges, BAF contributes to preventing their degeneration into cGAS-activating chromatin structures. This work helps better understand how the innate immune system detects mitotic defects.

## Author summary

The correct division of cells is essential for the development and maintenance of any organism. In this process, the cell’s nucleus containing chromosomes divides by mitosis. Errors in mitosis promote the emergence of cancers. Previous work showed that defects in mitosis can activate innate immune mechanisms that can promote the elimination of defective cells. This mechanism involves the protein cGAS that binds abnormal DNA and activates the protein STING that then induces the production of molecules capable of activating the immune system. However, the precise nature of the mitotic defects capable of triggering this response remained unclear. In this work, we provide evidence that threads of stretched chromosomes (chromatin bridges) that persist between two cells after defective mitosis are particularly potent activators of an innate immune response, compared with other types of cellular defects resulting from aberrant mitosis. We also show that a protein named BAF binds and stabilizes these chromatin bridges. When BAF is inactivated, chromatin bridges tend to break and become even stronger activators of the innate immune response. These findings help better understand how the immune system monitors cells against potentially harmful cells produced by defective mitosis. This knowledge could inform the improvement of immunotherapies against cancers.

## Introduction

Mitotic defects often lead to errors in chromosome segregation and genomic instability that can contribute to cancer progression [1,2]. Cancer cells often acquire structural nuclear defects [3,4]. Innate immune responses against foreign, mislocalized DNA or abnormal DNA-protein complexes can be co-opted to protect the organism against potentially hazardous cells resulting from mitotic defects [5,6]. Precisely which type of defective structures are detected and the nature of the downstream innate immune cascades being activated are incompletely understood.

The cyclic-GMP-AMP synthase (cGAS) - stimulator of interferon genes (STING) pathway is an innate immune response to abnormal DNA from endogenous or exogenous origins [6,7]. Upon binding to dsDNA, cGAS catalyzes the production of cyclic GMP-AMP (cGAMP), which binds the endoplasmic reticulum (ER) protein STING [8–13]. STING then translocates to the Golgi apparatus where it recruits and activates the inhibitor of nuclear factor kappa B kinase (IKK) complex and the IKK-related kinase TANK-binding kinase 1 (TBK1), leading to the phosphorylation and nuclear translocation of NFκB and Interferon Regulatory Factor 3 (IRF3), respectively. These transcription factors subsequently induce the expression of type I Interferons (INFs), proinflammatory cytokines and Interferon-Stimulated Genes (ISGs) [6,7]. These factors collaborate to activate and recruit leukocytes that induce localized inflammation and can eliminate problematic cells, which may be infected by a virus or potentially be cancerous. cGAS is activated by binding naked dsDNA but it is inhibited by its binding to nucleosomes [14–18]. As a result, although cGAS is present in the nucleus, it is not normally activated by chromatin, nor during mitosis when the nuclear envelope is disassembled. During mitosis, inhibitory phosphorylation of cGAS further contributes to preventing its activation [19,20].

Micronuclei are small nuclear structures in addition to the principal nucleus. They occur more frequently in cancer cells than in equivalent healthy cells [3]. Micronuclei can arise from mitotic errors. Anaphase bridges or lagging chromosomes can resolve into micronuclei in telophase (S1 Fig) [3]. Micronuclei are fragile; their nuclear envelope (NE) tends to break, exposing their DNA to cytoplasmic nucleases and cGAS [21]. Pioneering work presented evidence suggesting that micronuclei are potent activators of cGAS and its downstream innate immune response [22,23]. However, subsequent studies suggested that micronuclei are poor activators of cGAS [24–26]. One compelling study comparing various mitotic drug treatments, showed that chromatin bridges under tension were better activators of cGAS than micronuclei [25]. Another report indicated that mitochondrial DNA (mtDNA) generated concomitantly with micronuclei, was responsible for cGAS activation [26]. Therefore, the nature of the cellular defects capable of activating cGAS upon mitotic defects remains controversial or unclear.

Another source of post-mitotic micronuclei is defective nuclear reassembly from segregated chromosomes (S1 Fig) [3]. Barrier-to-Autointegration Factor (BAF) plays a crucial role in this process. This protein was identified as the most critical protein for preventing post-mitotic micronucleation in human cells [27]. BAF binds dsDNA, lamins and transmembrane proteins of the NE containing a LEM domain [28]. During mitotic entry, BAF phosphorylation triggers its release into the cytoplasm, facilitating NE breakdown [29]. During mitotic exit, BAF dephosphorylation by PP2A-Ankle2 allows BAF reloading onto segregated chromosomes and its recruitment of NE components [30,31]. Because BAF functions as a DNA-binding dimer, it cross-bridges chromosomes, thereby ensuring the formation of a single, spheroid nucleus [27]. In addition, BAF counteracts cGAS activation by competing with its binding to DNA [32].

The immunogenicity of post-mitotic nuclear defects could conceivably be exploited in the context of immunotherapy. Mitotic perturbations could be used to further enhance immunogenic nuclear defects in cancer cells, thereby boosting their detection and clearance by the immune system. However, the relative potencies of different types of perturbations in inducing this response are unclear. We compared the ability of chromosome segregation defects and nuclear reassembly defects to activate the cGAS-STING innate immune response, both alone and in combination. We found that while perturbing chromosome segregation is more potent than perturbing nuclear reassembly, combining both perturbations synergistically enhances the cGAS-STING response. Our results support the idea that cGAS recruitment on chromatin bridge remnants located in the perinuclear cytoplasm is a particularly strong activator of this response. We propose that BAF functions to stabilize chromatin bridges that emerge in anaphase, and that the loss of this function potentiates the cGAS-STING response.

## Results

### BAF depletion enhances a cGAS-STING dependent response induced by Reversine treatment‌‌

**Fig 1:**
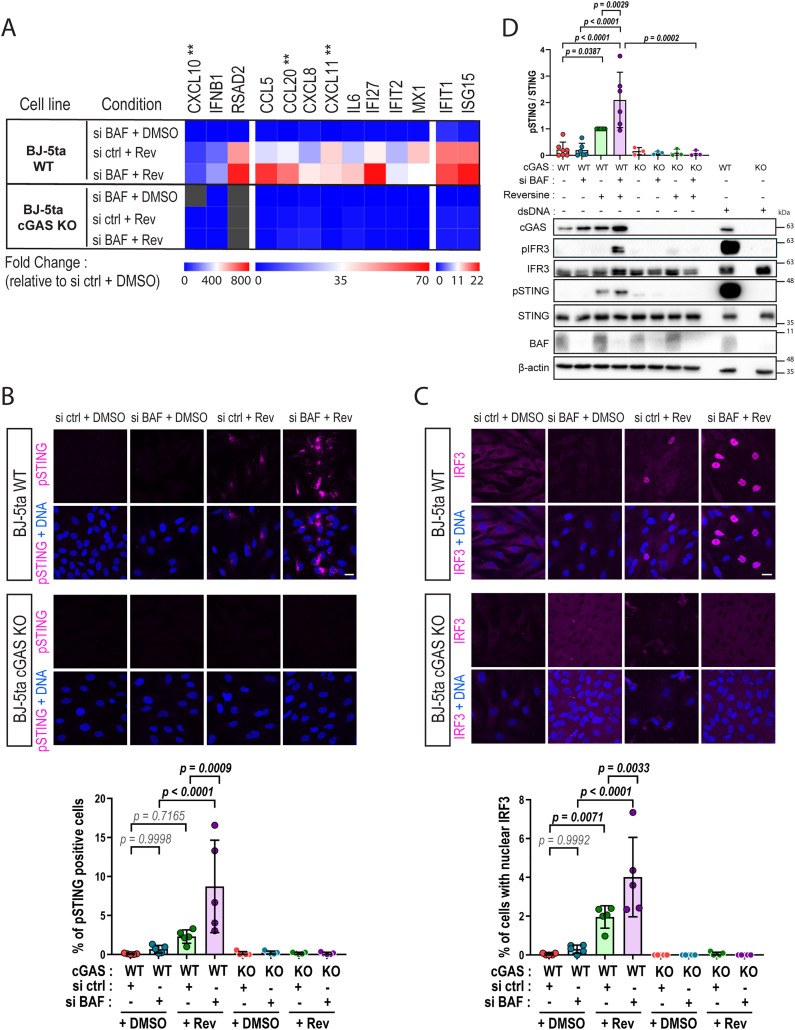
BAF depletion enhances a cGAS-STING dependent response induced by Reversine treatment. **A.** The expression of cytokines and ISGs was quantified by RT-qPCR after the indicated treatments in BJ-5ta cGAS WT or KO cells. For each factor measured, heatmap colors indicate the fold changes relative to the control (si ctrl + DMSO) according to the color scale underneath. Averages from 3 independent experiments (except for **:2 experiments; grey: not detected). **B, C.** Immunofluorescence to reveal pSTING (B) and IRF3 (C) after the indicated treatments in BJ-5ta cGAS WT or KO cells. Top: examples of images. Scale bars: 20 μm. Bottom: quantification of the percentages of cells showing a clear signal for pSTING or a nuclear enrichment of IRF3. Averages ± standard deviations (SD) from 5 independent experiments**.** A minimum of 1000 cells per condition were analyzed (One-way ANOVA shown). **D.** Western blots from BJ-5ta cGAS WT or KO cells treated as indicated. Cells not treated with Reversine received DMSO and cells not transfected with siRNA BAF received siRNA ctrl, except for cells treated with dsDNA which received nothing else. Note that pSTING is most intense in cells treated with Reversine and depleted of BAF. Top: quantification of pSTING/ STING signals ratios. Averages of at least 3 experiments (6 or 7 for cGAS WT cells) ±SD are shown. Values were normalized to 1 for the treatment with Reversine alone. p-values are from 2-way ANOVA.

We hypothesized that combining inhibition of the Spindle-Assembly Checkpoint (SAC) and BAF depletion, by enhancing post-mitotic micronucleation, would synergize in the induction of the cGAS-STING response (S1 Fig). Inhibition of the SAC using the MPS1 inhibitor Reversine results in anaphase bridges and lagging chromosomes that often become micronuclei during telophase [3,33,34]. On the other hand, BAF prevents post-mitotic micronucleation resulting from defective nuclear reassembly [27]. To test these perturbations, we used BJ-5ta cells, hTERT-immortalized fibroblasts, which have an active cGAS-STING pathway [25]. We used RT-qPCR to measure the expression of several IRF3- and NFκB-dependent type I IFNs, cytokines, ISGs and proinflammatory factors. We found that MPS1 inhibition induced a significant increase in the expression of several cytokines and ISGs (Fig 1A). This result is consistent with previous reports, which show that MPS1 inhibition with multiple compounds activates the cGAS-STING pathway [25,35]. By contrast, while the loss of BAF was previously shown to activate cGAS-STING [32,36,37], we detected only a minor activation of proinflammatory transcription when BAF was depleted alone. Interestingly, combining both perturbations enhanced the transcriptional response of most proinflammatory factors tested (Fig 1A). Similar results were obtained in MDA-MB-231 breast cancer cells (S2 Fig). In these cells, we also tested two alternative MPS1 inhibitors, BAY1217389 [38] and CFI-402257/Luvixasertib [39], and obtained similar results, ruling out potential off-target effects of Reversine (S3 Fig).

In addition, we found that passage through mitosis is required for this response. We used the CDK1 inhibitor Ro3306 to arrest cells in G2 [40]. As a result, the transcriptional response to Reversine, BAF depletion or both was strongly abrogated (S4 Fig). This result indicates that the defects triggering the cGAS-dependent response to Reversine and BAF depletion originate in mitosis.

To test if the observed proinflammatory transcriptional response depends on cGAS, we used cGAS KO BJ-5ta cells. We found that the absence of cGAS almost completely abolished the transcription of the tested proinflammatory factors induced by Reversine treatment or BAF depletion or both simultaneously (Fig 1A). Similar results were obtained with MDA-MB-231 cells depleted of cGAS by siRNA (S2 Fig). In BJ-5ta cells, we also used immunofluorescence to monitor markers of cGAS activation. We found that Reversine treatment and BAF depletion synergistically increased Ser366-phosphorylated STING (pSTING, active form) and the nuclear translocation of IRF3 (Fig 1B-1C). Increases in pSTING and Ser396-phosphorylated IRF3 (pIRF3, active form) were also observed by Western blot (Figs 1D and S5A). Interestingly, levels of cGAS, which is itself an ISG [41] were also increased (Figs 1D and S5B). Moreover, pSTING, nuclear IRF3 and pIRF3 signals were strongly abrogated by the KO of cGAS (Fig 1B-1D and S5A). Overall, our results indicate that MPS1 inhibition and BAF depletion synergistically enhance a cGAS-dependent proinflammatory transcriptional response, consistent with our hypothesis (S1 Fig).

### BAF depletion does not enhance Reversine-induced micronuclei/ lobulated nuclei or their recruitment of cGAS

**Fig 2:**
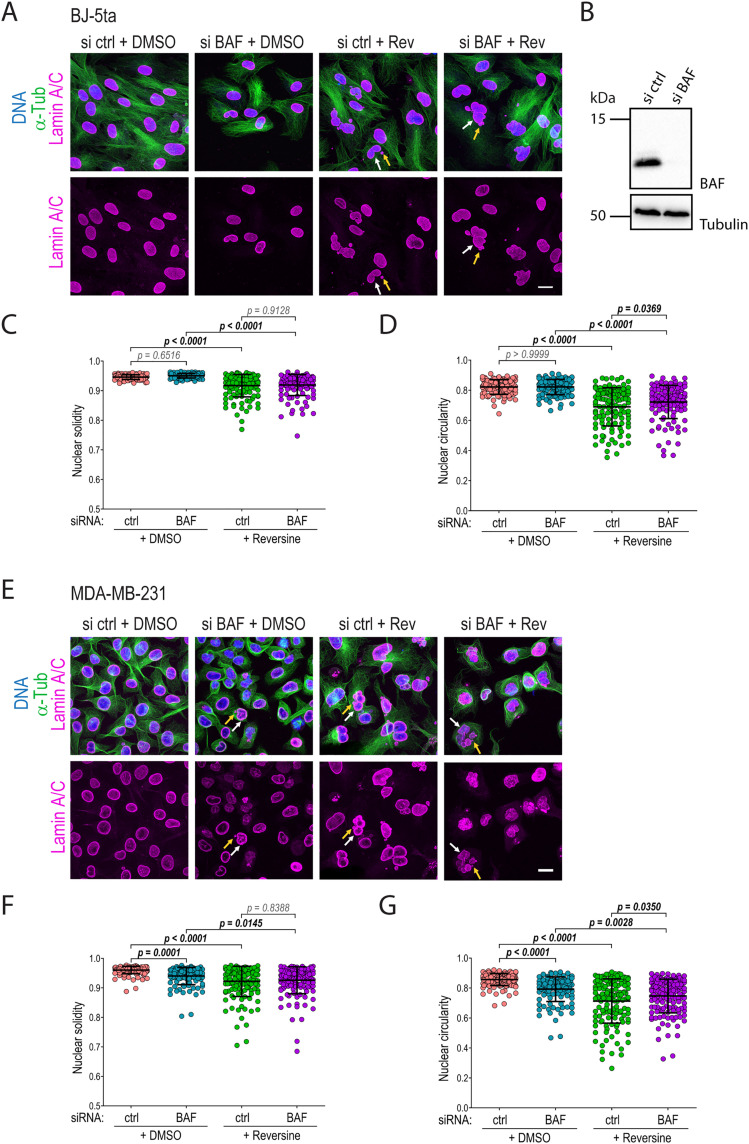
BAF depletion does not enhance Reversine-induced nuclear defects. BJ-5ta (**A**) or MDA-MB-231 (**E**) cells were analyzed by immunofluorescence to reveal structural nuclear defect (arrows). Examples of micronuclei (yellow arrows) and lobulated nuclei (white arrows) are shown. Scale bars: 20 μm. **B.** BAF depletion was verified by Western blot 4 days after siRNA transfection of BJ-5ta cells. Nuclear solidity (**C**, **F**) and nuclear circularity (**D**, **G**) were measured from cells treated as indicated from images as in A and **E.** Between 103 and 146 cells were analyzed for each condition. Averages ±SD and p-values from one-way ANOVA are shown.

According to our model, the increase in cGAS-STING response that we observed after combining Reversine treatment with BAF depletion would be due to an increase in post-mitotic micronucleation. In BJ-5ta cells, Reversine treatment resulted in frequent micronucleation and lobulated nuclei (Fig 2A). To quantify these defects, we measured nuclear solidity (see Materials & Methods). Nuclear circularity was also measured as an additional metric of nuclear structural aberration although it is not a measurement of micronucleation or lobulation. Indeed, nuclear solidity and circularity were both decreased upon Reversine treatment. Surprisingly, BAF depletion alone did not result in similar defects in BJ-5ta cells, nor did it increase these defects in combination with Reversine (Fig 2A-2D). Therefore, the cGAS-STING response activated above does not correlate with micronucleation/ lobulation in BJ-5ta cells, contrary to our hypothesis. Similar nuclear defects were obtained in cGAS KO cells, indicating that the lack of type I IFN, ISGs and proinflammatory cytokine genes transcription in these cells was not due to a failure to develop micronucleation/ lobulation (S6 Fig).

We obtained similar results in MDA-MB-231 (Fig 2E-2G) and HeLa cancer cells (S7 Fig). However, in these cell lines, BAF depletion alone resulted in nuclear defects, as quantified by solidity and circularity, unlike in BJ-5ta cells. The difference may be due to the observed faster doubling time of the two cancer cell lines compared with non-transformed fibroblasts, potentially helping the penetrance of the BAF depletion phenotype. Nevertheless, as in BJ-5ta cells, micronucleation/ lobulation was not further increased when BAF depletion was combined with Reversine treatment in MDA-MB-231 or HeLa cells. In fact, nuclear defects tended to decrease after both perturbations, possibly because BAF-depleted cells proliferated more slowly, thus impacting the incidence of post-mitotic phenotypes. These results indicate that combining SAC inhibition and BAF depletion does not enhance micronucleation/ lobulation, suggesting that the enhancement in the cGAS-STING response we observed is due to a distinct type of defect. Moreover, we found that treatment of BJ-5ta cells with 30 nM Taxol, although it resulted in strong micronucleation/lobulation, induced almost no proinflammatory transcriptional response (S8 Fig).

**Fig 3:**
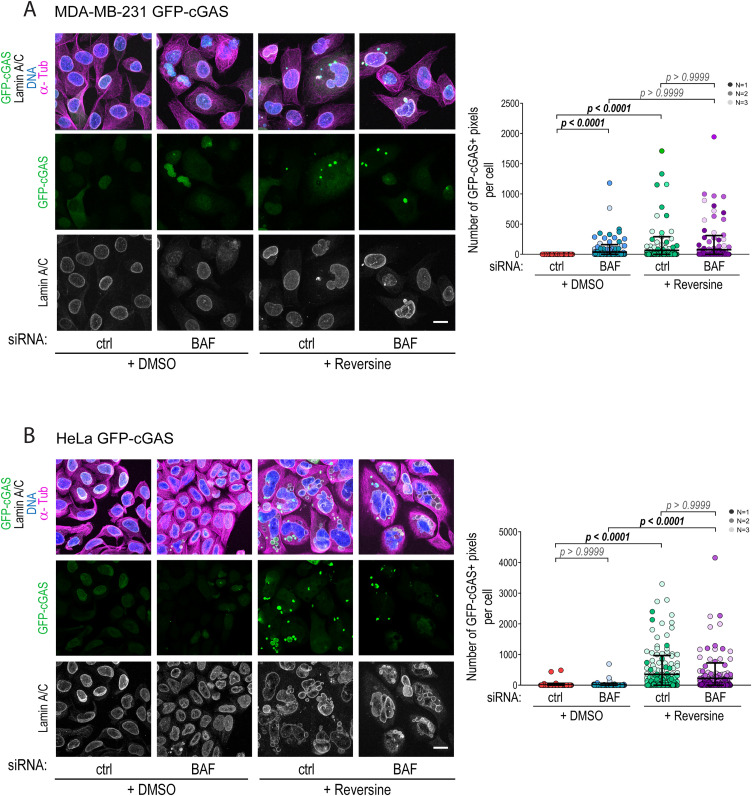
BAF depletion does not enhance the recruitment of cGAS on Reversine-induced defective nuclei. MDA-MB-231 cells (**A**) and HeLa cells (**B**) expressing GFP-cGAS were treated as indicated and analyzed by immunofluorescence. Left: examples of images. Scale bars: 20 μm. Right: Quantification of GFP-cGAS+ pixels of intensity above a fixed threshold. Averages ±SD from 3 independent experiments for which value sets (3 colors) were pooled on a same graph**.** More than 50 cells per condition per experiment were analyzed (Kruskal-Wallis test shown).

Nevertheless, we examined the possibility that the recruitment of cGAS to micronucleated or lobulated nuclei was enhanced by combining SAC inhibition and BAF depletion. To test it, we used MDA-MB-231 cells expressing GFP-cGAS, a fusion protein previously used to characterize the localization dynamics and interactions of cGAS [42]. In addition, our GFP-cGAS contained point mutations to make it catalytically inactive (E225A/D227A, to avoid potential artifacts due to a gain of function) [43]. We found that Reversine treatment and, to a lower degree BAF depletion, induced the recruitment of GFP-cGAS to micronuclei and lobulated nuclei. However, BAF depletion did not enhance the overall GFP-cGAS recruitment to nuclear structures induced by Reversine treatment (Fig 3A). Similar results were obtained in HeLa cells (Fig 3B). Together, our results suggest that the overall recruitment of GFP-cGAS to chromatin alone is not a good indicator of cGAS-STING activation in response to post-mitotic nuclear defects.

### Cytoplasmic DNA is not the main activator of cGAS-STING upon mitotic perturbations

**Fig 4:**
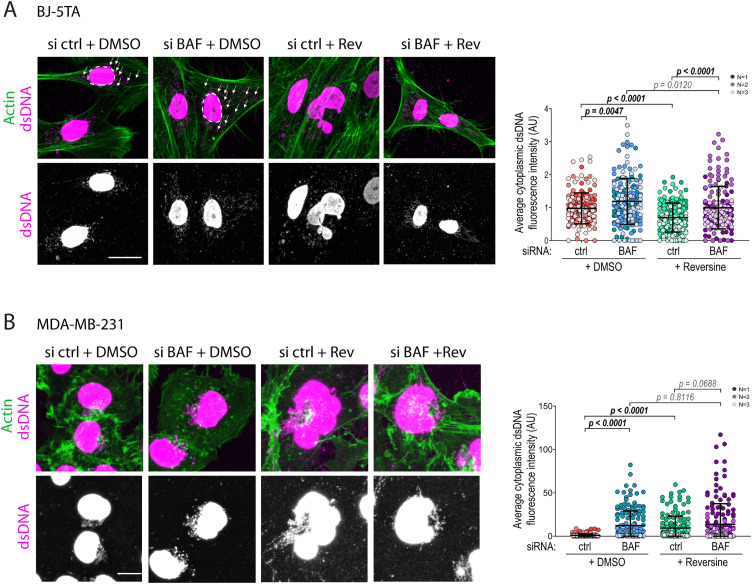
Quantification of cytoplasmic dsDNA upon mitotic perturbations. Immunofluorescence was done in BJ-5ta (**A**) and MDA-MB-231 (**B**) cells treated as indicated. Left: examples of images. Arrows: cytoDNA foci. Scale bars: 20 μm. Right: quantifications of average fluorescence intensities of dsDNA in the cytoplasm (outside of dotted line in example images). Averages ±SD from 3 independent experiments for which value sets (3 colors) were pooled on a same graph**.** More than 50 cells per condition per experiment were analyzed. In each experiment, values were normalized by setting the average value for si ctrl + DMSO to one. p-values are from one-way ANOVA.

The apparent uncoupling between the observed micronucleation/lobulation and the cGAS-STING dependent proinflammatory transcriptional induction suggested that this response was not due to these nuclear defects. We therefore examined the possibility that cytoplasmic DNA (cytoDNA) fragments could be responsible for the transcriptional response. Immunofluorescence against dsDNA revealed that BAF depletion led to a minor increase in cytoDNA in BJ-5ta cells. However, inactivation of the SAC with Reversine did not increase cytoDNA levels, with or without BAF depletion in BJ-5ta cells (Fig 4A). We obtained somewhat different results in MDA-MB-231 cells, where both BAF depletion and Reversine treatment increased cytoDNA. However, combining BAF RNAi and Reversine did not further increase cytoDNA levels (Fig 4B). We do not know why results differ between the two cell lines tested, but we surmise that the complex karyotype of MDA-MB-231 cells (between 52 and 68 chromosomes) may sensitize them to the production of cytoDNA. In any case, for both BJ-5ta and MDA-MB-231 cells, we found a flagrant lack of correlation between the increases in cytoDNA levels and activation of the cGAS-STING pathway (Fig 4, compare with Figs 1 and S2). In both cell lines, combining both perturbations did not further increase cytoDNA. These results suggest that cytoDNA, whether from nuclear or mitochondrial origin, does not contribute in a major way to the activation of cGAS-STING that we observed after perturbations of mitosis.

### BAF depletion modifies cGAS-positive chromatin bridges induced by Reversine

**Fig 5:**
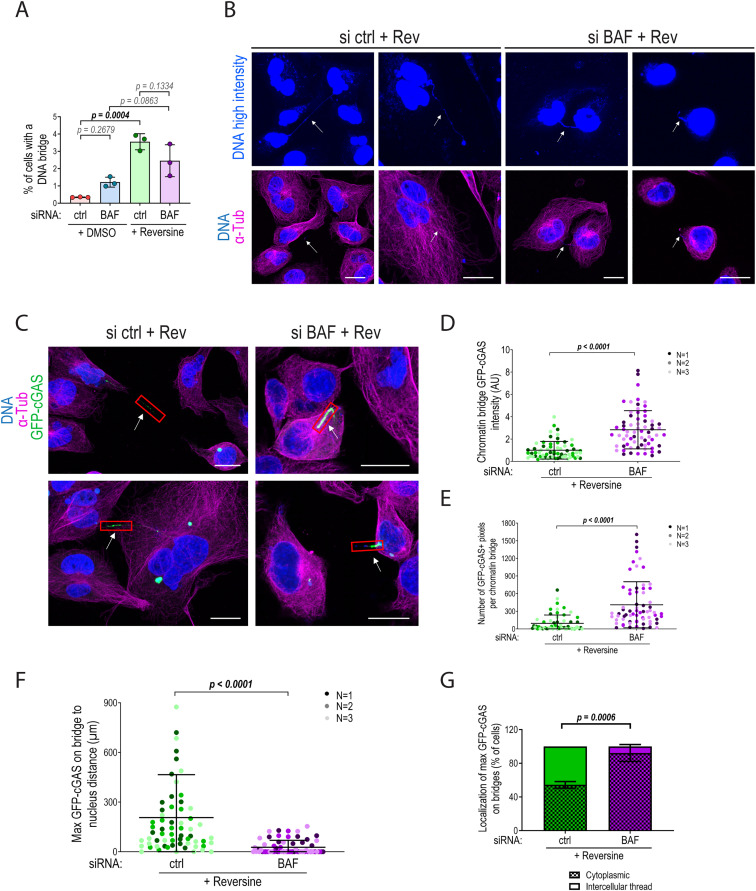
BAF depletion modifies cGAS-positive chromatin bridges induced by Reversine. **A.** The frequency of CBs/bridge remnants is not increased by the combination of Reversine treatment and BAF depletion, compared with single treatments. MDA-MB-231 cells expressing GFP-cGAS were used. Averages ±SD from 3 independent experiments. More than 500 cells were analyzed per condition (one-way ANOVA shown). **B.** Examples of immunofluorescence images revealing CBs or bridge remnants (arrows). **C.** Examples of immunofluorescence images revealing GFP-cGAS recruitment on CBs or bridge remnants (red boxes). **D-E.** BAF depletion increases GFP-cGAS maximal intensity (D) and the number of GFP-cGAS+ pixels of intensity above a fixed threshold (E) on CBs/bridge remnants in Reversine-treated cells. **F.** BAF depletion results in a shorter distance between maximal GFP-cGAS localization on CBs/bridge remnants and the nearest connected nucleus, in Reversine-treated cells. **G.** BAF depletion increases the frequency of cells showing their maximal GFP-cGAS localization on CBs/bridge remnants in the cytoplasm upon Reversine treatment. Quantification of cytoplasmic vs non-cytoplasmic (intercellular thread) maximal GFP-cGAS localization on CBs/bridge remnants after the indicated treatments. For D-F, averages ±SD from 3 independent experiments are shown and the 3 value sets (3 colors) were pooled on a same graph**.** Between 18 and 26 bridges per condition per experiment were analyzed (Student’s unpaired T test shown). Scale bars: 20 μm.

While the ability of micronuclei to induce a cGAS-STING response has been a subject of controversy, one study proposed that anaphase chromatin bridges (CBs) are effective triggers of this response [25]. SAC inhibition, as occurs with the MPS1 inhibitor Reversine, is known to induce CBs as anaphase occurs before chromosomes are all correctly attached to the spindle [33]. These CBs can persist until the next interphase, connecting daughter cells [33,44,45]. Using MDA-MB-231 cells expressing GFP-cGAS, we found that Reversine treatment resulted in frequent CBs, including apparent remnants of broken bridges (Fig 5A-5C). GFP-cGAS localized to CBs (Fig 5C), where it persisted for hours, appearing more stable than the nuclear pool of GFP-cGAS known to be subjected to degradation in G1 (S9 Fig) [46]. However, combining BAF depletion with Reversine treatment did not further increase the frequency of CBs compared to either treatment alone (Fig 5A). These results suggest that BAF depletion enhances the cGAS-dependent transcriptional response to SAC inhibition by a mechanism other than a simple increase in the frequency of CBs.

Although the frequency of CBs was not enhanced, we observed phenotypic differences. We found that the depletion of BAF strongly increased the recruitment of GFP-cGAS at CBs and bridge remnants induced after Reversine treatment (Figs 5C-5E and S10). In addition, we found that the region of maximal GFP-cGAS intensity was located differently. In cells treated with Reversine alone, GFP-cGAS tended to be localized inside a long thin CB, away from the nucleus (Fig 5C, 5F-5G). Similar results were obtained by probing endogenous cGAS by immunofluorescence (Fig S11). In approximately half of those cells, the region of most intense GFP-cGAS occurred in an intercellular thread, an area practically devoid of cytoplasm, away from the nucleus (Fig 5F-5G). By contrast, in cells treated with Reversine and depleted of BAF, GFP-cGAS tended to be most intense near the nucleus, in the cytoplasm of the cell body. In many of those cells, the most intense point of GFP-cGAS occurred on hook-like CB remnants within the bulk of the cytoplasm (Fig 5C). Similar results were obtained in BJ-5ta cells expressing GFP-cGAS (S12 Fig). Our results are consistent with the idea that CBs are responsible for the activation of cGAS-STING [25]. Moreover, our results further suggest that BAF modifies CBs, thereby altering their ability to activate the cGAS-STING response.

### Ankle2-dependent BAF recruitment stabilizes chromatin bridges and limits cGAS activation

**Fig 6:**
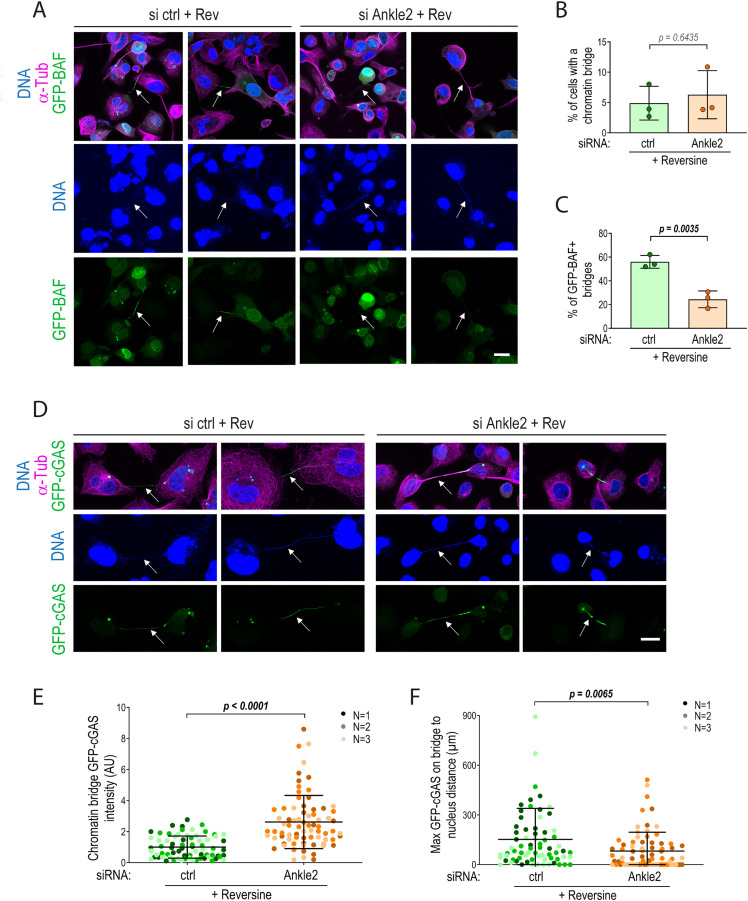
BAF is recruited to chromatin bridges in an Ankle2-dependent manner. **A.** Examples of immunofluorescence images revealing Reversine-induced CBs (arrows) that are GFP-BAF-positive (si ctrl) or -negative (si Ankle2). MDA-MB-231 cells expressing GFP-BAF were used. **B.** The frequency of Reversine-induced CBs is not increased by the depletion of Ankle2. Averages ±SD from 3 independent experiments. More than 500 cells were analyzed per condition (Student’s unpaired T test shown). **C.** Quantification of the percentage of CBs displaying clear GFP-BAF signal in cells depleted of Ankle2 or not. Averages ±SD from 3 independent experiments. Between 100 and 128 cells per condition per experiment were analyzed (Student’s unpaired T test shown). **D.** Immunofluorescence on MDA-MB-231 cells stably expressing GFP-cGAS and treated as indicated. **E.** Ankle2 depletion increases GFP-cGAS maximal intensity on CBs/bridge remnants in Reversine-treated cells. **F.** Ankle2 depletion results in a shorter distance between maximal GFP-cGAS localization on CBs/bridge remnants and the nearest connected nucleus, in Reversine-treated cells. For E, F, averages ±SD from 3 independent experiments for which value sets (3 colors) were pooled on a same graph**.** Between 22 and 26 bridges per condition per experiment were analyzed (Student’s unpaired T test shown). Scale bars: 20 μm.

Based on the above results, we hypothesized that the depletion of BAF results in a fragilization of CBs resulting from SAC inhibition. The resulting broken CB remnants appear to then retract towards the cytoplasm, where they recruit cGAS more efficiently. To test if BAF is recruited to CBs during anaphase, we used MDA-MB-231 cells expressing GFP-BAF. We found that GFP-BAF was strongly recruited to Reversine-induced CBs (Fig 6A-6C). This finding is consistent with a potential role for BAF in stabilizing CBs.

**Fig 7:**
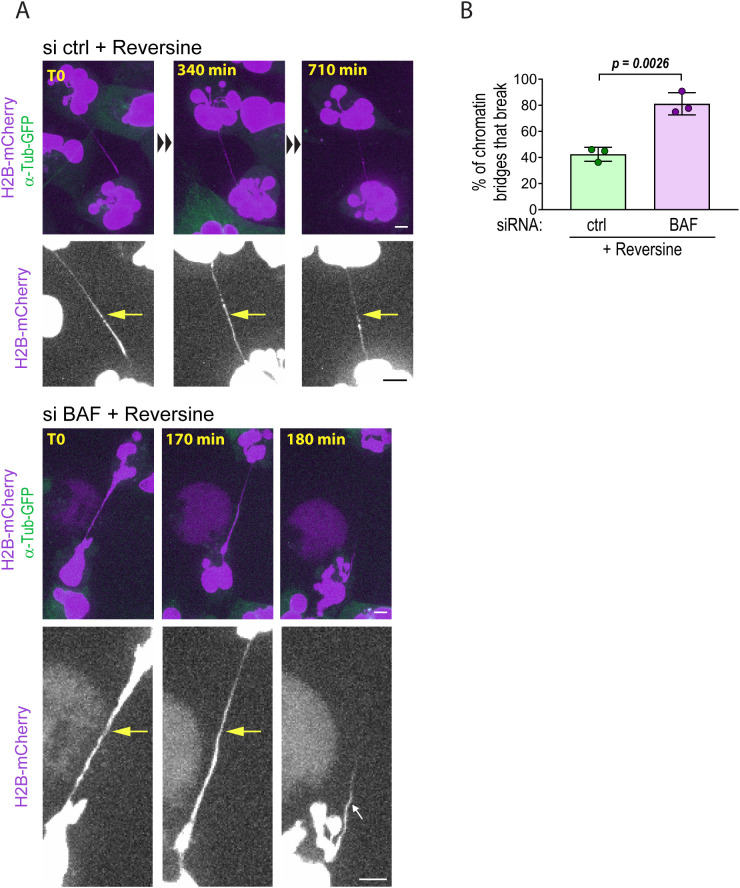
BAF stabilizes chromatin bridges. **A.** MDA-MB-231 cells expressing H2B-mCherry and α-Tubulin-GFP were filmed after treatments as indicated. Examples of images from videos are shown. Yellow arrows indicate intact CBs and white arrows indicate bridge remnants. Scale bar: 10 μm. **B.** Quantification of the percentage of cells showing CBs that broke during the 12 h of filming. Averages ±SD from 3 independent experiments**.** Between 11 and 22 CBs per condition per experiment were analyzed (Student’s unpaired T test shown).

To test if BAF depletion destabilizes CBs, we filmed MDA-MB-231 cells expressing H2B-mCherry and α-Tubulin-GFP treated with Reversine and depleted of BAF or not. We followed the CBs that were visible throughout the 12 hours of filming. We scored the percentage of bridges that broke within this time frame (Fig 7 and S1-S2 Videos). We found that more CBs broke in cells depleted of BAF compared to cells treated with Reversine only. We obtained similar results in HeLa cells expressing H2B-mCherry and α-Tubulin-GFP (S13 Fig and S3-S4 Videos). These results confirm that BAF promotes the stability of CBs.

To test if the enhancement of the cGAS-dependent transcriptional response depends specifically on the loss of BAF recruitment to CBs during anaphase, we depleted Ankle2. This protein is required for the PP2A-dependent dephosphorylation of BAF that allows the binding of BAF to DNA during anaphase [30,31,47]. We found that Ankle2 depletion markedly decreased the recruitment of GFP-BAF on CBs (Fig 6A, 6C), without decreasing the incidence of CBs themselves (Fig 6B). As for the depletion of BAF, depletion of Ankle2 led to a marked increase in GFP-cGAS intensity at Reversine-induced CBs (Fig 6D-6E). Moreover, the GFP-cGAS bearing CBs or remnants were located closer to nuclei when Ankle2 was depleted (Fig 6F).

**Fig 8:**
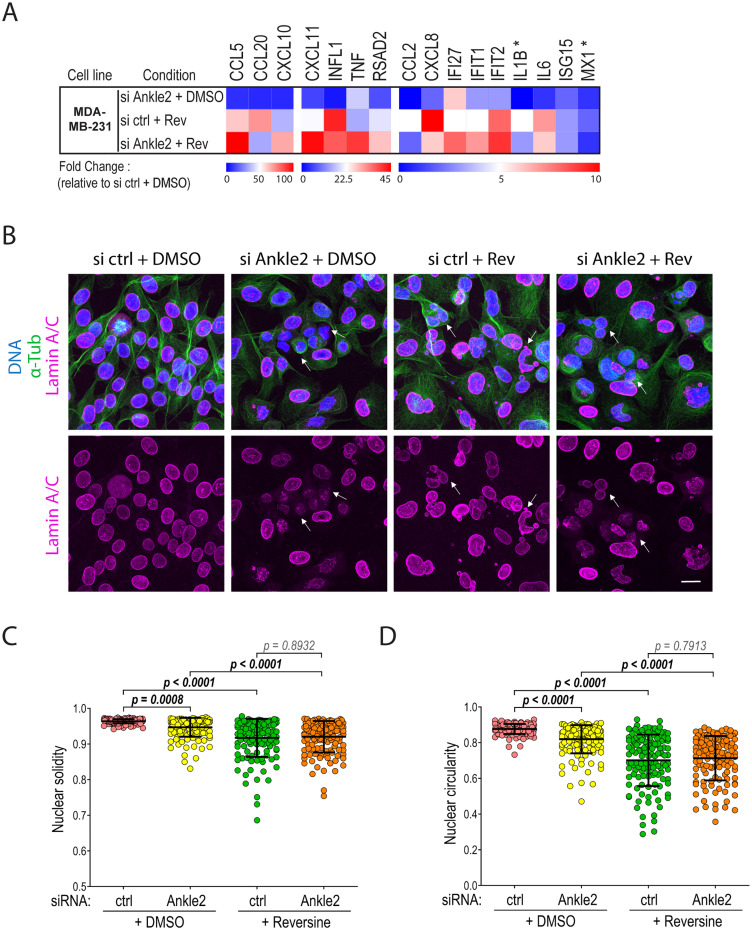
Ankle2 depletion enhances the proinflammatory transcriptional response to Reversine treatment without enhancing micronucleation/lobulation. **A.** The expression of cytokines and ISGs was quantified by RT-qPCR after the indicated treatments of MDA-MB-231 cells. For each factor measured, heatmap colors indicate the fold changes relative to the control (si ctrl + DMSO) according to the color scale underneath. Averages from 3 independent experiments (except for *:1 experiment). **B-D.** Ankle2 depletion does not enhance micronucleation/lobulation upon Reversine treatment. MDA-MB-231 cells were analyzed by immunofluorescence to reveal structural nuclear defects (arrows). Scale bar: 20 μm. **(B)**. Nuclear solidity (C) and nuclear circularity (D) were measured from cells treated as indicated. For C-D, between 126 and 140 cells per condition were analyzed. Averages ±SD and p-values from one-way ANOVA are shown.

**Fig 9:**
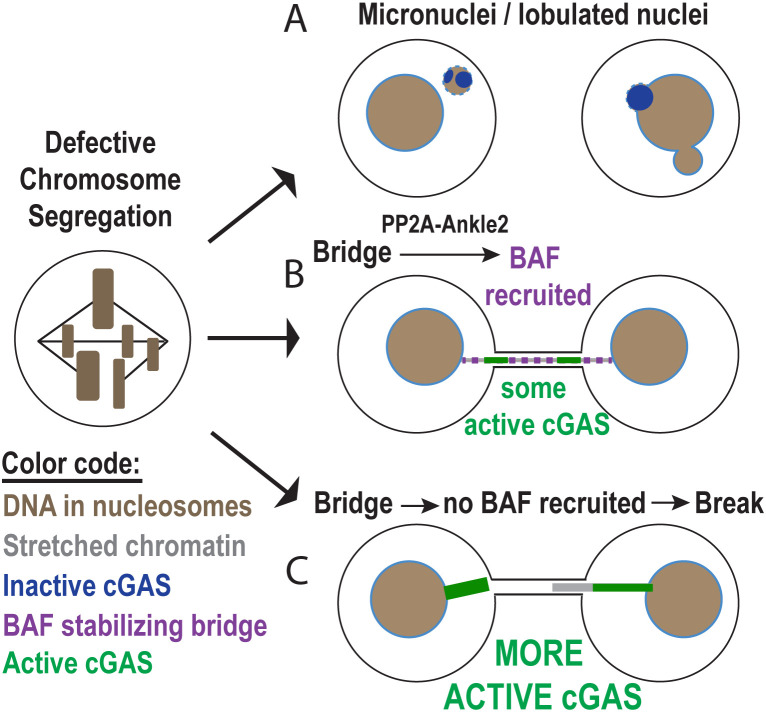
Model for the role of BAF in the modulation of cGAS-STING signaling in response to chromatin bridges. **A.** Defective chromosome segregation can lead to micronuclei and lobulated nuclei where cGAS is recruited but is inhibited by nucleosomes. **B**. Defective chromosome segregation can also lead to chromatin bridges (CBs) where chromatin is stretched and can recruit and activate cGAS. However, BAF is recruited to these CBs in a PP2A-Ankle2-dependent manner to stabilize CBs in thin intercellular threads. BAF also competes with cGAS for DNA binding on CBs. **C**. If BAF cannot be recruited to CBs, they tend to break, resulting in cytoplasmic bridge remnants that are more potent activators of cGAS.

We then tested if the reduction of BAF at CBs upon Ankle2 depletion correlates with an enhancement of the cGAS-dependent transcriptional response. Similarly to the depletion of BAF, depletion of Ankle2 alone in MDA-MB-231 cells generally resulted in a modest increase in the expression of the proinflammatory factors examined. Combining Ankle2 depletion with Reversine treatment resulted in a further increase in the expression of most factors (Fig 8A). This effect was not as strong as that observed upon BAF depletion, possibly because some BAF can still be recruited to CBs after Ankle2 depletion (Fig 6C). Nevertheless, the observed increase in proinflammatory transcription did not correlate with an overall increase in micronucleation/lobulation measured by solidity and circularity (Fig 8B-8D). These results suggest that the PP2A-Ankle2 dependent recruitment of BAF on CBs promotes their stabilization and is associated with a downregulation of their ability to induce a cGAS-STING transcriptional response (Fig 9).

## Discussion

The interplay between mitosis and innate immunity to self-DNA has been the subject of many recent studies [21,48–50]. However, the precise nature of post-mitotic defects capable of triggering an innate immune response remained unclear. Studies in mice implicated micronuclei as activators of cGAS [22,23]. However, subsequent studies using human cells provided compelling evidence indicating that micronuclei were poor activators of cGAS-STING [24–26]. In addition, one study provided evidence that CBs were better activators of this response [25]. CytoDNA, potentially leaked out of malformed nuclei or from mitochondria, is another possible contributor that we have examined [6,7]. Our results support the idea that CBs are particularly potent activators of the cGAS-STING response. We also discovered that BAF modifies the dynamics of CBs, thereby also impacting the cGAS-STING response.

The previous study by the Mitchison lab used various drugs affecting mitosis and cytokinesis to treat BJ-5ta fibroblasts and measured paracrine activation of STING through secreted cGAMP in THP1 monocytes containing an ISRE-dependent luciferase reporter. A clear correlation was observed between the incidence of stretched CBs and paracrine cGAS-STING activation [25]. Here, we provide further support for their conclusions using immortalized BJ-5ta fibroblasts and MDA-MB-231 triple-negative breast cancer cells. We induced mitotic defects by perturbing the SAC and nuclear reassembly. We demonstrated that STING activation, as monitored by Western blot and immunofluorescence analysis, the nuclear import of IRF3 and its downstream proinflammatory transcription correlated with the intensity of cGAS on CBs. While Flynn et al relied on a co-culture assay to measure paracrine cGAS-STING activation, here we measured cGAS-STING activation directly in the perturbed cells. Our results in BJ-5ta and MDA-MB-231 cells are similar, suggesting that the activation of cGAS-STING by CBs may be a process that is common to multiple types of human cells, transformed or not. Of note, while Flynn et al observed CBs that correlated with cGAS-STING activation in response to docetaxel, we did not observe a marked increase in CBs or a clear activation of proinflammatory transcription upon treatment of BJ-5ta cells with the analogous compound Taxol (S8D-S8E Fig).

While cGAS is recruited to micronuclei and lobulated nuclei, the incidence of these structures or the intensity of cGAS recruited on them does not correlate with STING activation. This should not be surprising because nuclear DNA is generally packaged in nucleosomes, and because cGAS is inhibited when bound to nucleosomes [14–18,51]. In contrast, CBs are subjected to pulling forces that may be capable of unravelling DNA from nucleosomes (Fig 9) [44,52]. The cGAS recruited on the putative nucleosome-free DNA would be expected to be highly active, accounting for the correlation between the incidence of cGAS-positive CBs and STING activation. However, we did not observe a strict correlation between GFP-cGAS-positive CBs and pSTING on a single-cell basis by immunofluorescence. While CBs typically break down within hours [44], the duration of cGAS-STING proinflammatory signaling persistence after its trigger is removed remains unclear. While remnants of CBs can be discerned as hook-like structures in some cells, they may reintegrate the nucleus or become closely juxtaposed to it, making it impossible to know that a pSTING-positive cell once had a CB. In addition, cGAS-STING signaling functions largely in a paracrine manner, through the secretion of cGAMP [53]. Thus, STING may become activated in cells that were once neighbours of cells with CBs. Importantly, despite all the correlative evidence presented here and in the Flynn et al (2021) study, a direct demonstration that pools of cGAS on CBs are more active than pools of cGAS on micronuclei or lobulated nuclei is still lacking. It remains possible that some cGAS recruited on micronuclei or lobulated nuclei is activated if chromatin integrity is altered at those sites.

We identified BAF as a potent regulator of CB-associated cGAS-STING signaling. We initially chose to inactivate BAF because of its reported role in preventing micronucleation during post-mitotic nuclear reassembly [27]. While we observed a modest increase in lobulated nuclei, STING activation and cGAS-dependent proinflammatory transcription after BAF depletion alone, it is only in the context of SAC inhibition that we observed a strong effect of BAF depletion. Similar observations were recently reported by another group [54]. Interestingly, we discovered that BAF stabilizes CBs. The CB-bound pool of BAF is recruited in a manner that depends on Ankle2, a PP2A regulatory subunit that specifically promotes BAF dephosphorylation [30,31,47,55]. Without BAF recruitment, CBs break more frequently, resulting in chromatin structures that recruit more cGAS. In addition, the pool of cGAS on these CB remnants tends to reside more proximally to the nucleus in the cell body, where the Golgi and ER (on which STING functions), as well as the bulk of the cytoplasm, reside. Compared to CBs that are largely constrained in thin intercellular threads, cGAS-STING signaling from CB remnants in the cell body may be facilitated by a better diffusion of cGAS, its substrates (nucleotides) and/or its product cGAMP.

The mechanism by which BAF stabilizes CBs in cells is unclear. It was recently demonstrated that BAF, in complex with LEM2, forms a phase-separated hydrogel that coats DNA and enhances its resistance to pulling forces *in vitro*, a mechanism that is likely to operate on CBs [56]. Another possibility is that BAF may antagonize cytoplasmic nucleases such as TREX1 and ANKLE1 that digest and cut DNA in CBs [44,57–59]. Interestingly, a study in *Drosophila* has shown a role for BAF in promoting the reintegration of broken or resolved CBs into the nucleus [60]. If a similar function is at play in human cells, CB remnants, failing to reintegrate the nucleus, may remain more capable of activating cGAS in BAF-depleted cells. In addition to the specific function of BAF at CBs, the ability of BAF to directly compete with cGAS for binding to DNA may contribute to its regulation of CB-induced cGAS-STING signaling. While this function of BAF was demonstrated in the context of sporadic damage to the nuclear envelope in interphase [32], our results show that cells must undergo mitosis to trigger cGAS-STING signaling in response to SAC inhibition and BAF depletion. Nevertheless, the higher levels of GFP-cGAS at CBs when BAF is depleted is likely due in part to the competition between the two proteins for DNA binding.

We examined the possibility that cytoDNA could contribute to cGAS-STING activation in the presence of mitotic defects. An increase in cytoDNA was seen after BAF depletion and likely results from damage to the nuclear envelope, which can allow the leakage of nuclear DNA fragments into the cytoplasm [61,62]. Intriguingly, in MDA-MB-231 cells, we also observed an increase in cytoDNA upon SAC inhibition. It is possible that DNA fragments are released from the shredding of CBs by cytoplasmic nucleases. Finally, we cannot rule out the possibility that mitotic defects could indirectly lead to a leakage of mtDNA into the cytoplasm, contributing to cGAS activation. In any case, the poor correlation we observed between levels of cytoDNA vs STING activation and downstream transcription suggests that cytoDNA is not a major contributor to cGAS-STING signaling in response to mitotic defects.

It will be important to investigate the novel cellular role of BAF in the stabilization of CBs after mitosis. This function of BAF may promote genome integrity by allowing the correct, damage-free resolution of CBs before they break. Such a function would add to its regulation of DNA repair proteins [28]. Given the accumulating evidence pointing at CBs as particularly potent activators of cGAS-STING signaling, their pharmacological induction should be considered as a potential avenue for enhancing the efficiency of immunotherapy against tumors that have an active cGAS-STING pathway. This approach may be particularly effective in tumors showing a high propensity to chromosome segregation defects. The induction of CBs in tumors is already possible using MPS1 inhibitors in clinical trials [63,64]. While BAF itself is not readily druggable, the enzyme required for its dephosphorylation and recruitment to DNA after mitosis, PP2A, is an enzyme that can be inhibited [65,66]. However, inhibiting all forms of PP2A would have pleiotropic effects on cellular functions. The capability of selectively inactivating PP2A-Ankle2, which is specialized for BAF regulation, would be desirable to specifically interfere with BAF function to enhance cGAS-STING signaling in the context of immunotherapy.

## Materials and methods

### DNA constructs

GFP-cGAS and GFP-BAF lentiviral vectors were generated by Gateway cloning. BAF and cGAS coding sequences flanked with attB sites were cloned into the entry vector pDONR221 and then recombined into pLVpuro-CMV-Nterm-eGFP expression vector for lentivirus production and stable transduction in cells (Addgene plasmid #122848). The plasmid containing the BAF sequence flanked with attB sites was custom made by Biobasic (hBAF-att #100013339-UMON272 or #240803HT1931–1). The sequence of catalytically inactive cGAS was amplified from pBabe-cGAS(E225A/D227A)-mCherry2 (gift from Kyle Roux, U. South Dakota), using the cloning oligos attB1-cGAS-F (GGGGACAAGTTTGTACAAAAAAGCAGGCTTAATGCAGCCTTGGCACGGAAAG) and attB2-cGAS-stop-R (GGGGACCACTTTGTACAAGAAAGCTGGGTCCTAAAATTCATCAAAAACTGGAAACTCATTGTTTCTTTCATATTC) (Integrated DNA Technologies).

### Cell lines and cell culture

All cell lines were cultured at 37°C with 5% CO_2_ in DMEM medium (Wisent) supplemented with 10% fetal bovine serum (FBS, Wisent). MDA-MB-231 cells were a gift from Geneviève Deblois (IRIC-Université de Montréal). BJ-5ta WT and BJ-5ta cGAS KO were a gift from Tim Mitchison (Harvard University). MDA-MB-231 and HeLa cells expressing H2B-mCherry and α-tubulin-GFP were given by Beth Weaver (U. of Wisconsin-Madison) and Daniel Gerlich (IMBA Vienna), respectively. Stable cell lines overexpressing either GFP-BAF or GFP-cGAS were generated by lentiviral infections and selection for resistance to puromycin. For lentivirus production, HEK293T cells were transfected with pLVpuro-CMV-GFP-cGAS or pLVpuro-CMV-GFP-BAF along with psPAX2 (Addgene #12260) and pCMV-VSV-G (Addgene #8454) plasmids using polyethyleneimine. MDA-MB-231, BJ-5ta and HeLa cells were transfected with the virus using protamine sulfate and selected using 0.5 µg/mL, 0.7 µg/mL or 1 µg/mL puromycin in the medium respectively. Cells were maintained in DMEM medium under the same conditions. Cells were kept at low passages for all experiments.

### RNA interference and drug treatments

siRNA transfections were performed using Lipofectamine RNAiMAX transfection reagent (Invitrogen #13778075) according to the manufacturers’ instructions. Cells were transfected with non-targeting siRNA (as control) or siRNA against BAF, Ankle2 or cGAS protein two times for 48h each. SMARTpool ON-TARGETplus human BAF siRNA (#L-011536-02-0010), human Ankle2 siRNA (#L-181819-00-0005), human cGAS siRNA (#L-015607-02-0005) and ON-TARGETplus Non-targeting siRNA (#D-001810-10-20) Pool were purchased from Dharmacon/Horizon Discovery. Cells were treated with Reversine (Cayman Chemical Co #10004412) at 500 nM, Taxol (MedChemExpress Co #YH-B0015) at 30 nM, BAY-1217389 (MedChemExpress Co #HY-12859) at 20 nM, CFI-402257 (MedChemExpress Co #HY-101340) at 750 nM or DMSO only as control immediately after the second siRNA transfection, before being analyzed by immunofluorescence or Western blotting 48h later. To arrest cells in G2, cells were treated with Ro3306 (Tocris Bioscience #4181) at 10 µM for 48h following the second transfection.

### Western blotting

For results shown in Figs 2, S2, S3 and S7, cells were collected by trypsinization and washed in PBS containing protease inhibitors (1 mM PMSF, 10 μg/mL Aprotinin and 10 μg/mL Leupeptin). Cells were then lysed in lysis buffer (50 mM Tris-HCl pH 7.5, 150 mM NaCl, 0.2% Triton, 10% glycerol) containing the same protease inhibitors for 10 min at 4°C. For results shown in Fig 1, cells in 6-well plates were washed once with PBS and flash frozen in liquid nitrogen. Proteins from whole cell extracts (WCE) were obtained by resuspending cells in Triton X-100 lysis buffer (50 mM Tris-HCl, pH 7.4, 150 mM NaCl, 50 mM NaF, 5 mM EDTA, 40 mM β-glycerophosphate, 1% Triton X-100, 10% glycerol, 1 mM sodium orthovanadate, 1 mM pefabloc, 1 mM leupeptin, 1 mM pepstatin A, 1 mM aprotinin) for 30 min at 4°C. Lysates were clarified by centrifugation at 20,000 × *g* for 15 min. Proteins from WCE were quantified using BCA or Bradford protein assay (BioRad) according to the manufacturer’s protocol before mixing with Laemmli buffer at 95°C for 5 mins. Samples were run on a standard SDS-PAGE gel, and proteins were then transferred onto nitrocellulose membranes. Membranes were blocked using TBS + 0.1% Tween 20 (TBS-T) containing 5% powder milk for 1h. They were then incubated overnight at 4°C with primary antibodies diluted in blocking solution except for pSTING and pIRF3 antibodies which were diluted in TBS-T containing 1% BSA. After 3 washes with TBS-T, membranes were incubated for 40–60 minutes with secondary antibodies coupled with peroxidase. After 3 washes with TBS-T, membranes were incubated with Clarity Western ECL Substrates (Bio-Rad #170–5061) and imaged using the ChemiDoc MP system. The following primary antibodies were used: α-tubulin DM1A (#7291 Abcam, 1:1000), STING -D2P2F- (mAb#1364 Cell Signaling, 1:1000), pSTING -D8K6H- (mAb#40818 Cell Signaling, 1:1000), IRF3 -D6I4C- XP (mAb#11904 Cell Signaling, 1:1000), pIRF3 (Cell Signaling #4947, 1:1000), cGAS -D1D3G- (mAb#15102, Cell Signaling, 1:500), Ankle2 [N1N3] (#GTX120698 GeneTex, 1:1000), BAF (#129074–1001 Abcam, 1:1000). Anti-rabbit or anti-mouse antibodies coupled with peroxidase were used as secondary antibodies (respectively #111-035-008 and #115-035-003, Jackson ImmunoResearch, 1:5000). Band intensities were quantified by the software Bio-Rad Image Lab Version 6.1.

### Immunofluorescence

For Immunofluorescence, 60 000 cells were plated on Poly-L-lysine-coated coverslips (Sigma Aldrich) for 48h. Cells were fixed with 4% formaldehyde in PHEM buffer (60 mM Pipes, 25 mM HEPES, 10 mM EGTA, 4 mM MgSO_4_, pH 6.9) for 20 min, then blocked and permeabilized with PHEM + BSA 2% + triton 0.1% for 1 h before incubation for 2 h with primary antibody diluted in PHEM + BSA 2%. Cells were washed 3 times for 5 min with TBS-tween 20 0.1% and incubated with secondary antibody and DAPI (Sigma Aldrich, 1:1000) for 1.5h. After 3 washes with TBS-Tween 20 0.1%, cells were mounted on glass coverslips using Vectashield Mounting Medium (#H-1000 Vector laboratories). The following primary antibodies were used: Lamin A/C (#L-1293 Sigma Aldrich, 1:1000), Phospho-STING 5Ser366) (D8K6H) (mAb#40818 Cell Signaling, 1:200), IRF3 -D6I4C- XP (mAb#11904 Cell signaling, 1:400), α-tubulin DM1A (#ab7291 Abcam, 1:500), dsDNA Marker (#sc-58749 Santa Cruz Biotechnology, 1:100), cGAS -D1D3G- (mAb#15102, Cell Signaling, 1:200). To detect Actin, we used Alexa Fluor 488 phalloidin (#A12379 Invitrogen, 1:1000). The following secondary antibodies were used: Alexa Fluor 488-Conjugated AffiniPure Goat Anti-Mouse IgG (115-545-166, Jackson Immunoresearch, 1:200), Alexa Fluor 647 goat anti-Rabit IgG (#A21244 Invitrogen, 1:200), Cy3-conjugated AffiniPure Goat Anti-Mouse IgG (115-165-166, Jackson Immunoresearch, 1:200), Goat anti-Rabbit IgG (#A27039 Invitrogen, 1:200), Texas RedR-X goat anti-mouse IgG (#T6390 Invitrogen, 1:200).

### Microscopy

Imaging of fixed cells by immunofluorescence was done on a LSM700 or LSM880 laser scanning confocal microscope (Zeiss) using a 40X or 63X oil objective. Images were scanned at a resolution of 512 x 512 pixels or 1024 x 1024 pixels at 7 frames per second for image quantification or Fig production respectively.

Chromatin bridges (Figs 5A, 6B and S8E) were manually scored on an Axio microscope using a 63X or 100X oil objective. pSTING positive cells (Fig 1B) and cells with nuclear IRF3 (Fig 1C) were also scored this way. For live cell imaging, cells were plated with indicated treatments on film chambers (Lab-Tek #082123-8-2) and incubated for 24 h before imaging. Cells were imaged each 10 minutes for 12 h within an incubation chamber at 37°C with 5% CO_2_ using the Spinning Disk confocal system (Yokogawa CSU-X1 5000) mounted on a fluorescence microscope (Zeiss Axio Observer Z1). Maximal intensity projections of those films were performed on Zen software to score bridge breakage events (Figs 7B and S13B) and extract bridge images shown as examples in Figs 7A and S13A.

### Fluorescence quantifications

GFP-cGAS recruitment was measured using SUM projections of confocal images (made using Fiji software). The number of GFP-cGAS+ pixels for each cell was measured in Zen software using the α-tubulin staining to delineate the outline of each cell within which the number of GFP-cGAS+ pixels was measured. The fluorescence intensity threshold used to consider a pixel GFP-cGAS + was determined for each independent experiment based on si ctrl DMSO conditions in which no cGAS recruitment was observed on nuclear structures. For GFP-cGAS recruitment to chromatin bridges, the average intensity of the most intense GFP-cGAS region (rectangle of fixed size: 468 pixel^2^) of each bridge was measured in Zen using SUM projections performed in Fiji. The number of GFP-cGAS+ pixels on chromatin bridges was also measured as above, but by restricting measurements to areas tightly surrounding bridges. The distance between this high GFP-cGAS intensity region and the closest nucleus from which the bridge originates was also measured on Zen (Fig S10). For all GFP-cGAS measurements, the background was subtracted from all measures for each cell or bridge.

For measurements of cytoplasmic DNA, pixels in the area corresponding to the nuclear DAPI staining were first set to zero intensity. From the resulting image, the average intensity of fluorescence of the entire cell was taken to reflect the levels of cytoplasmic DNA.

### Circularity and solidity measurements

Solidity and circularity measurements were made using the “analyze particles” tool in ImageJ on maximum intensity projections of DAPI staining of cells. The same “mask parameters”, chosen to maximize the detection and visualisation of micronuclei and lobulated nuclei were applied for each cell within a same condition. Solidity is defined as the ratio of the measured area of the nucleus to that of a bounding convex shape [67,68]. Circularity is a measurement of how round a shape is compared to a perfect circle and is defined as 4π x area/ perimeter^2^. The shape analyzed was restricted to the principal nucleus with immediately juxtaposed micronuclei, and thus micronuclei appearing separated from the main shape were not included, contributing to an underestimation of solidity and circularity defects in some cells.

### RT-qPCR

Cells were harvested by trypsinization and cell pellets were washed in PBS containing protease inhibitors (1 mM PMSF, 10 μg/mL Aprotinin and 10 μg/mL Leupeptin). Pellets were kept, when needed, at -80°C for 2 days maximum before RNA extraction. RNA was extracted using the RNeasy Mini Kit (#74104 Qiagen) and the corresponding instructions. RNA was eluted in 30 µl of TE buffer and RT-qPCR analyses were performed by IRIC’s genomic platform as previously described [69]. Primers used for each of the factors are listed in S1 Table. ACTB and HPRT were used as internal controls. Fold changes for each factor were expressed as compared to the si ctrl + DMSO condition. Heat maps were designed using the “Morpheus” tool from the Broad Institute.

### Figures and statistical analyses

Graphs and statistical analyses were performed using GraphPad software. All results are expressed as mean ± SD unless otherwise indicated. Sample size and tests used are specified in each figure legend. Data distribution was assumed to be normal (except for Fig 3A and 3B), but this was not formally tested. All raw numerical data shown in the figures are available in S1 File.

## Supporting information

S1 Fig Initial model for cGAS-STING activation in response to post-mitotic nuclear defects. Post-mitotic nuclear defects including micronuclei and lobulated nuclei can result from errors in chromosome segregation or nuclear reassembly. We hypothesized that combining perturbations in both processes may enhance these defects and the associated activation of cGAS-STING signaling.
(TIF)

S2 Fig BAF depletion enhances a cGAS-STING dependent response induced by Reversine treatment in MDA-MB-231 cells. **A.** The expression of cytokines and ISGs was quantified by RT-qPCR after the indicated treatments in MDA-MB-231 cells. For each factor measured, heatmap colors indicate the fold changes relative to the control (si ctrl + DMSO) according to the color scale underneath. Averages from 4 independent experiments (except for ***:3 experiments). **B.** Western blots showing the depletion of BAF and cGAS in cells after the indicated treatments.
(TIF)

S3 Fig BAF depletion enhances a proinflammatory transcriptional response induced by two alternative MPS1 inhibitors in MDA-MB-231. **A.** Examples of images of immunofluorescence in cells treated with DMSO (control), BAY-1217389 (20 nM) or CFI-402257 (750 nM). Chromatin bridges (arrowheads), micronuclei (yellow arrows) and lobulated nuclei (white arrows) are shown. Scale bars: 10 μm. **B.** The expression of cytokines and ISGs was quantified by RT-qPCR after the indicated treatments in MDA-MB-231 cells. For each factor measured, heatmap colors indicate the fold changes relative to the control (si ctrl + DMSO) according to the color scale underneath. **C.** Western blots showing the depletion of BAF in cells after the indicated treatments.
(TIF)

S4 Fig The cGAS-STING response to Reversine and BAF depletion requires passage through mitosis. **A.** The expression of cytokines and ISGs was quantified by RT-qPCR from BJ-5ta cells treated as indicated. For each factor measured, heatmap colors indicate the fold changes relative to the control (si ctrl + DMSO) according to the color scale underneath. **B.** Quantification of the mitotic index from DAPI staining indicating that the CDK1 inhibitor Ro3306 blocked mitotic entry. For A and B, representative results of two independent experiments are shown.
(TIF)

S5 Fig Additional quantification of Western blot signals from Fig 1D. **A.** Ratios of pIRF3/ IRF3 signals. Averages of at least 3 experiments (6 or 7 for cGAS WT cells) ±SD are shown. **B.** Ratios of cGAS/ β-actin signals. Averages of 3 experiments ±SD are shown. p-values are from 2-way ANOVA.
(TIF)

S6 Fig Measurements of nuclear defects in BJ-5ta cGAS KO cells. Nuclear solidity (A) and nuclear circularity (B) were measured from cells treated as indicated and processed by immunofluorescence as in Fig 2. Between 111 and 115 cells per condition were analyzed. Averages ±SD and p-values from one-way ANOVA are shown.
(TIF)

S7 Fig BAF depletion does not enhance Reversine-induced nuclear defects in HeLa cells. **A.** Western blotting showing the depletion of BAF 4 days after transfection with siRNA. **B.** HeLa cells were analyzed by immunofluorescence to reveal structural nuclear defect (arrows). Scale bars: 20 μm. **C-D.** Nuclear solidity (C) and nuclear circularity (D) were measured from HeLa cells expressing GFP-cGAS and treated as indicated from images as in B. Between 96 and 131 cells per condition were analyzed. Averages ±SD and p-values from one-way ANOVA are shown.
(TIF)

S8 Fig Taxol induces severe nuclear defects but little proinflammatory transcriptional response in BJ-5ta cells. **A.** Immunofluorescence on cells treated as indicated to reveal nuclear defects (arrows). Taxol was used at 30 nM. Scale bars: 20 μm. **B-C.** Nuclear solidity (B) and nuclear circularity (C) were measured in cells treated as indicated. Averages ±SD from 2 independent experiments for which value sets (2 colors) were pooled on a same graph. More than 100 cells per condition per experiment were analyzed (Student’s unpaired T test shown). **D.** The expression of cytokines and ISGs was quantified by RT-qPCR after the indicated treatments in BJ-5ta WT cells. For each factor measured, heatmap colors indicate the fold changes relative to the control (DMSO) according to the color scale underneath. Averages from 2 independent experiments. Note that the nuclear defects following Taxol treatment are quantitatively similar to those obtained after Reversine treatment (Fig 2) but that the transcriptional response is much weaker (Fig 1). All cells were transfected with siRNA ctrl. **E.** Taxol treatment of BJ-5ta cells does not induce a marked increase in CBs. Averages from 2 independent experiments ±SD are shown. More than 500 cells per condition per experiment were analyzed (Student’s unpaired T test shown).
(TIF)

S9 Fig GFP-cGAS localizes to CBs for several hours after mitosis. Two examples of time-lapse image series of MDA-MB-231 cells expressing GFP-cGAS and treated with Reversine are shown. GFP-cGAS remains strongly localized on post-mitotic CBs (arrows) for at least 10 hours while GFP-cGAS in the nucleus (arrowheads) decreases in intensity. Scale bars: 10 μm.
(TIF)

S10 Fig Method used to quantify the intensity and position of GFP-cGAS on chromatin bridges. Immunofluorescence image from MDA-MB-231 cells expressing GFP-cGAS. A box of a fixed shape and area (red rectangle) was placed around the point of highest intensity of GFP-cGAS on the CB or bridge remnant. The mean fluorescence intensity of the GFP-cGAS signal within the box was measured and the mean intensity of the background measured from an immediately adjacent box was subtracted. The distance between the maximal GFP-cGAS intensity on CBs and the nearest nucleus to which it is connected was measured as indicated by the red line. Scale bar: 20 μm.
(TIF)

S11 Fig BAF depletion modifies cGAS-positive chromatin bridges induced by Reversine (complement to Fig 5). **A.** Examples of immunofluorescence images revealing endogenous cGAS on CBs or bridge remnants (red boxes). Scale bars: 10 μm. **B.** BAF depletion increases cGAS maximal intensity on CBs/bridge remnants in Reversine-treated cells. **C.** BAF depletion results in a shorter distance between maximal cGAS localization on CBs/bridge remnants and the nearest connected nucleus in Reversine-treated cells. For B-C, averages ±SD from 3 independent experiments are shown. The 3 value sets (3 colors) were pooled on a same graph. Between 23 and 28 bridges per condition per experiment were analyzed (Student’s unpaired T test shown).
(TIF)

S12 Fig BAF depletion modifies cGAS-positive chromatin bridges induced by Reversine in BJ-5ta cells. **A.** Examples of immunofluorescence images revealing GFP-cGAS recruitment on CBs or bridge remnants (arrows). Scale bar: 20 μm. **B.** BAF depletion increases GFP-cGAS maximal intensity on bridges of Reversine-treated cells. **C.** BAF depletion results in a shorter distance between maximal GFP-cGAS localization on CBs/bridge remnants and the nearest connected nucleus in Reversine-treated cells. **D.** BAF depletion increases the frequency of cytoplasmic maximal GFP-cGAS localization on CBs/bridge remnants upon Reversine treatment. Quantification of cytoplasmic vs non-cytoplasmic (intercellular thread) maximal GFP-cGAS localization on CBs/bridge remnants after the indicated treatments. For B-D, Averages ±SD from 3 independent experiments are shown. In B and C, the 3 value sets (3 colors) were pooled on a same graph. Between 18 and 28 bridges per condition per experiment were analyzed (Student’s unpaired T test shown).
(TIF)

S13 Fig BAF stabilizes chromatin bridges. **A.** HeLa cells expressing H2B-mCherry and α-Tubulin-GFP were filmed after treatments as indicated. Examples of images from videos are shown. Yellow arrows indicate intact CBs and white arrows indicate bridge remnants. Scale bar: 10 μm. **B.** Quantification of the percentage of cells showing CBs that broke during the 12 h of filming. Averages ±SD from 3 independent experiments. Between 9 and 22 CBs per condition per experiment were analyzed (Student’s unpaired T test shown).
(TIF)

S1 Video MDA-MB-231 cells expressing H2B-mCherry and α-Tubulin-GFP and treated with siRNA ctrl  + Reversine (complement to Fig 7). Note that the chromatin bridge persists throughout the 12 h of filming. Scale bar: 5 μm. Speed: 2 frames per second.
(AVI)

S2 Video MDA-MB-231 cells expressing H2B-mCherry and α-Tubulin-GFP and treated with siRNA BAF + Reversine (complement to Fig 7). Note that the chromatin bridge breaks during the 12 h of filming. Scale bar: 5 μm. Speed: 2 frames per second.
(AVI)

S3 Video HeLa cells expressing H2B-mCherry and α-Tubulin-GFP and treated with siRNA ctrl + Reversine (complement to S13 Fig). Note that the chromatin bridge persists throughout the 12 h of filming. Scale bar: 6.7 μm. Speed: 10 frames per second.
(AVI)

S4 Video HeLa cells expressing H2B-mCherry and α-Tubulin-GFP and treated with siRNA BAF + Reversine (complement to S13 Fig). Note that the chromatin bridge breaks during the 12 h of filming. Scale bar: 5 μm. Speed: 10 frames per second.
(AVI)

S1 Table Primer oligonucleotides used for RT-qPCR analyses. (DOCX)

S1 File Numerical data for all figs‌‌. (XLSX)
