# Supplementary figures and images for: Barrier-to-autointegration factor protects against the cGAS-STING response to chromatin bridges
Source: PLoS Genet. 2026 Jun 3;22(6):e1012191. doi: 10.1371/journal.pgen.1012191 (PMC13258145; doi:10.1371/journal.pgen.1012191)

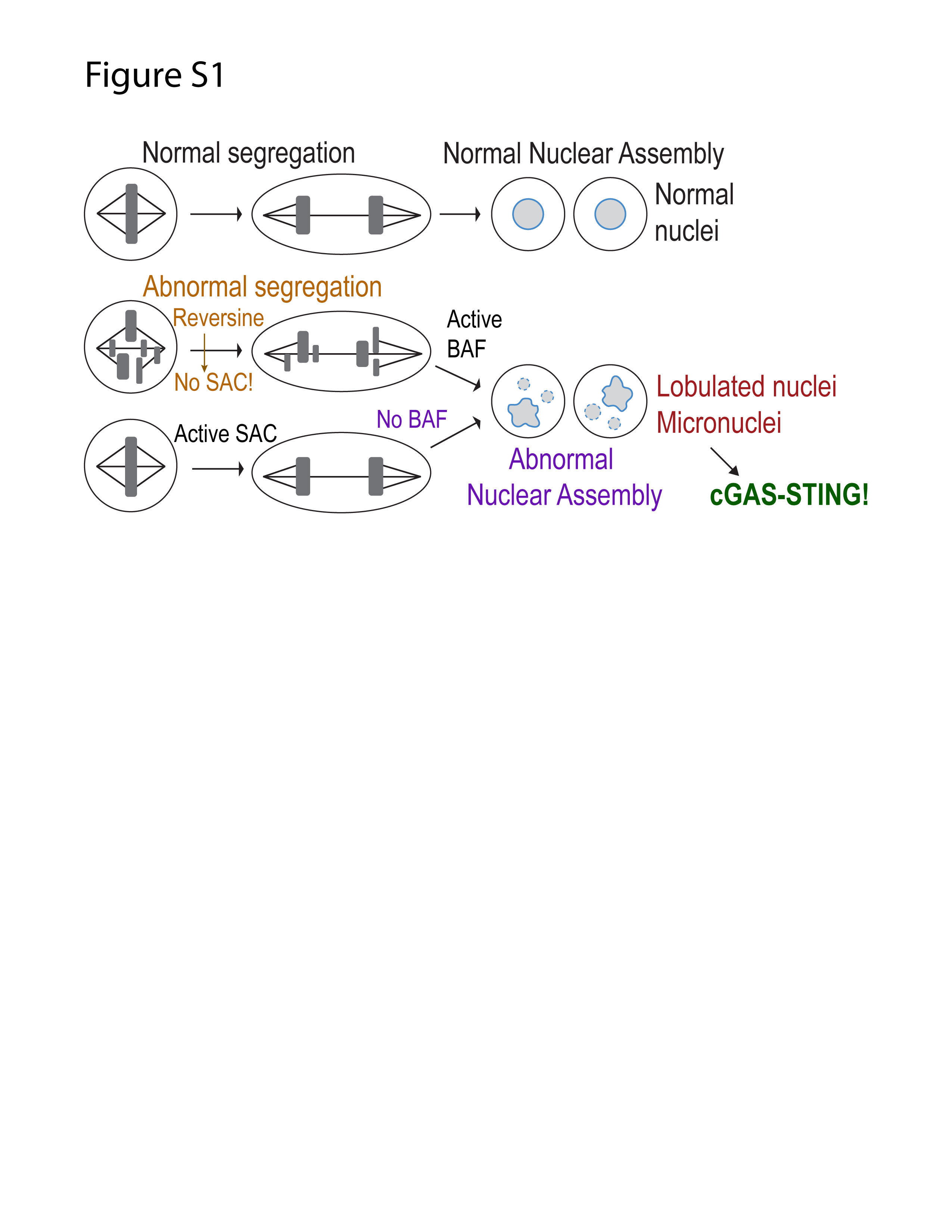

Supplement: S1 Fig — Post-mitotic nuclear defects including micronuclei and lobulated nuclei can result from errors in chromosome segregation or nuclear reassembly. We hypothesized that combining perturbations in both processes may enhance these defects and the associated activation of cGAS-STING signaling. (TIF) [file pgen.1012191.s001.tif]

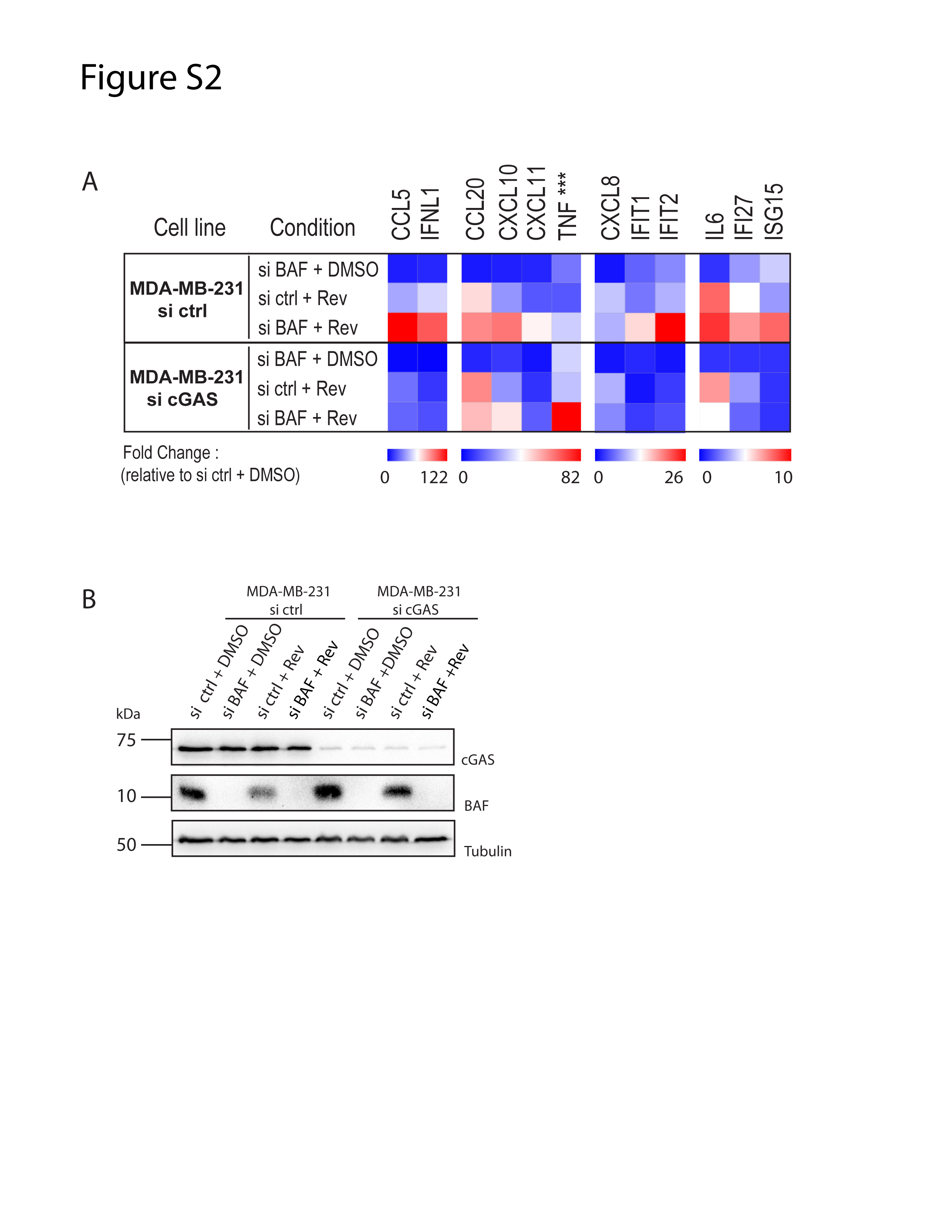

Supplement: S2 Fig — A. The expression of cytokines and ISGs was quantified by RT-qPCR after the indicated treatments in MDA-MB-231 cells. For each factor measured, heatmap colors indicate the fold changes relative to the control (si ctrl + DMSO) according to the color scale underneath. Averages from 4 independent experiments (except for ***:3 experiments). B. Western blots showing the depletion of BAF and cGAS in cells after the indicated treatments. (TIF) [file pgen.1012191.s002.tif]

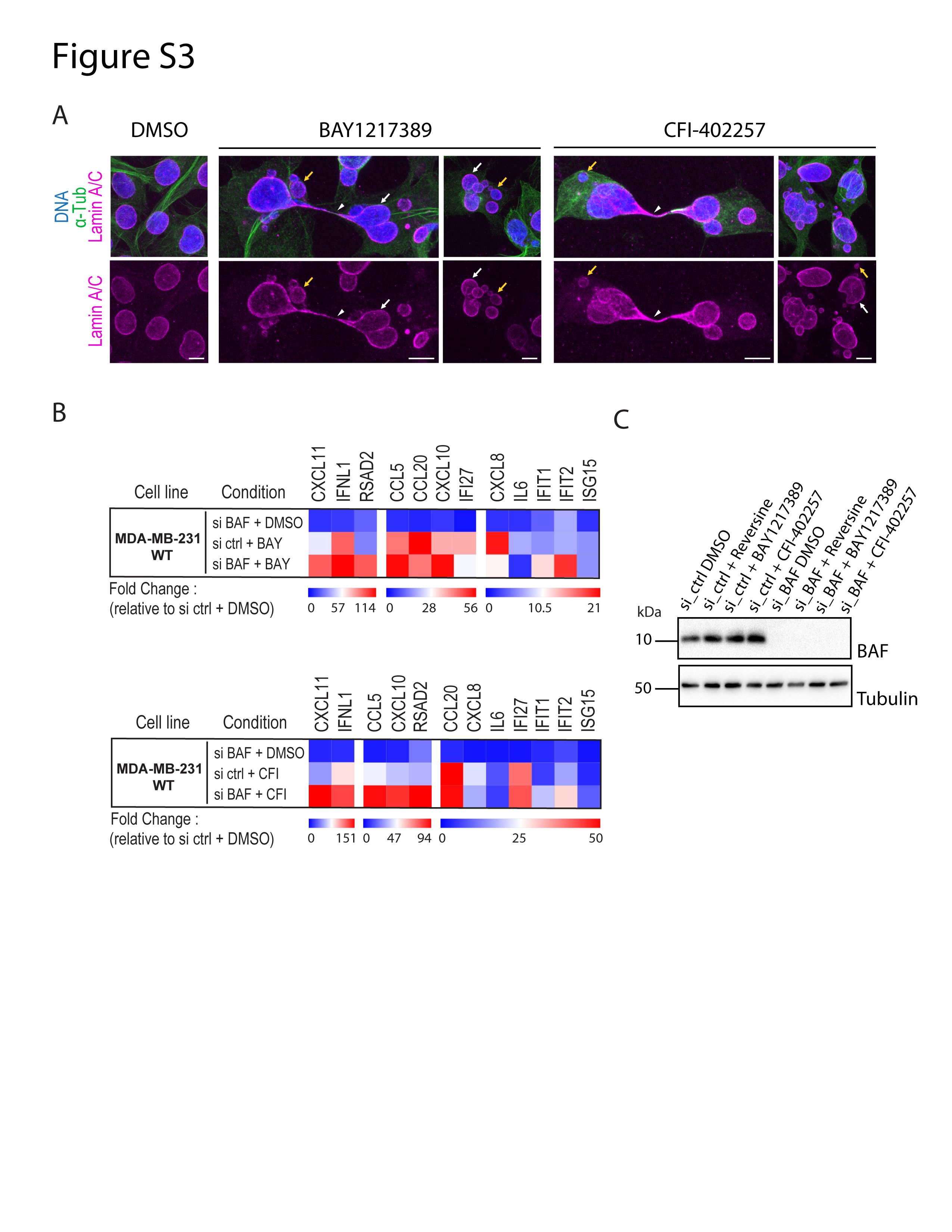

Supplement: S3 Fig — A. Examples of images of immunofluorescence in cells treated with DMSO (control), BAY-1217389 (20 nM) or CFI-402257 (750 nM). Chromatin bridges (arrowheads), micronuclei (yellow arrows) and lobulated nuclei (white arrows) are shown. Scale bars: 10 μm. B. The expression of cytokines and ISGs was quantified by RT-qPCR after the indicated treatments in MDA-MB-231 cells. For each factor measured, heatmap colors indicate the fold changes relative to the control (si ctrl + DMSO) according to the color scale underneath. C. Western blots showing the depletion of BAF in cells after the indicated treatments. (TIF) [file pgen.1012191.s003.tif]

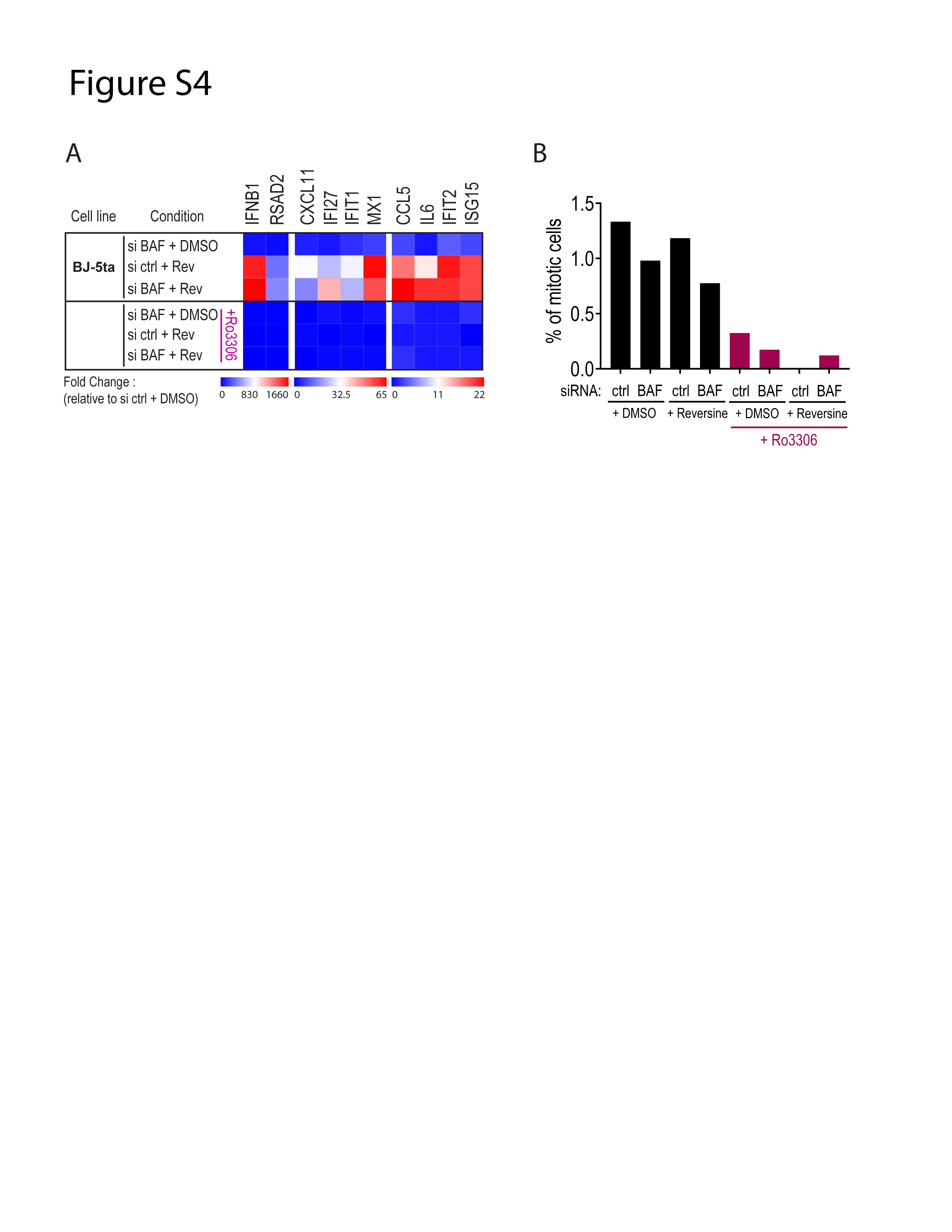

Supplement: S4 Fig — A. The expression of cytokines and ISGs was quantified by RT-qPCR from BJ-5ta cells treated as indicated. For each factor measured, heatmap colors indicate the fold changes relative to the control (si ctrl + DMSO) according to the color scale underneath. B. Quantification of the mitotic index from DAPI staining indicating that the CDK1 inhibitor Ro3306 blocked mitotic entry. For A and B, representative results of two independent experiments are shown. (TIF) [file pgen.1012191.s004.tif]

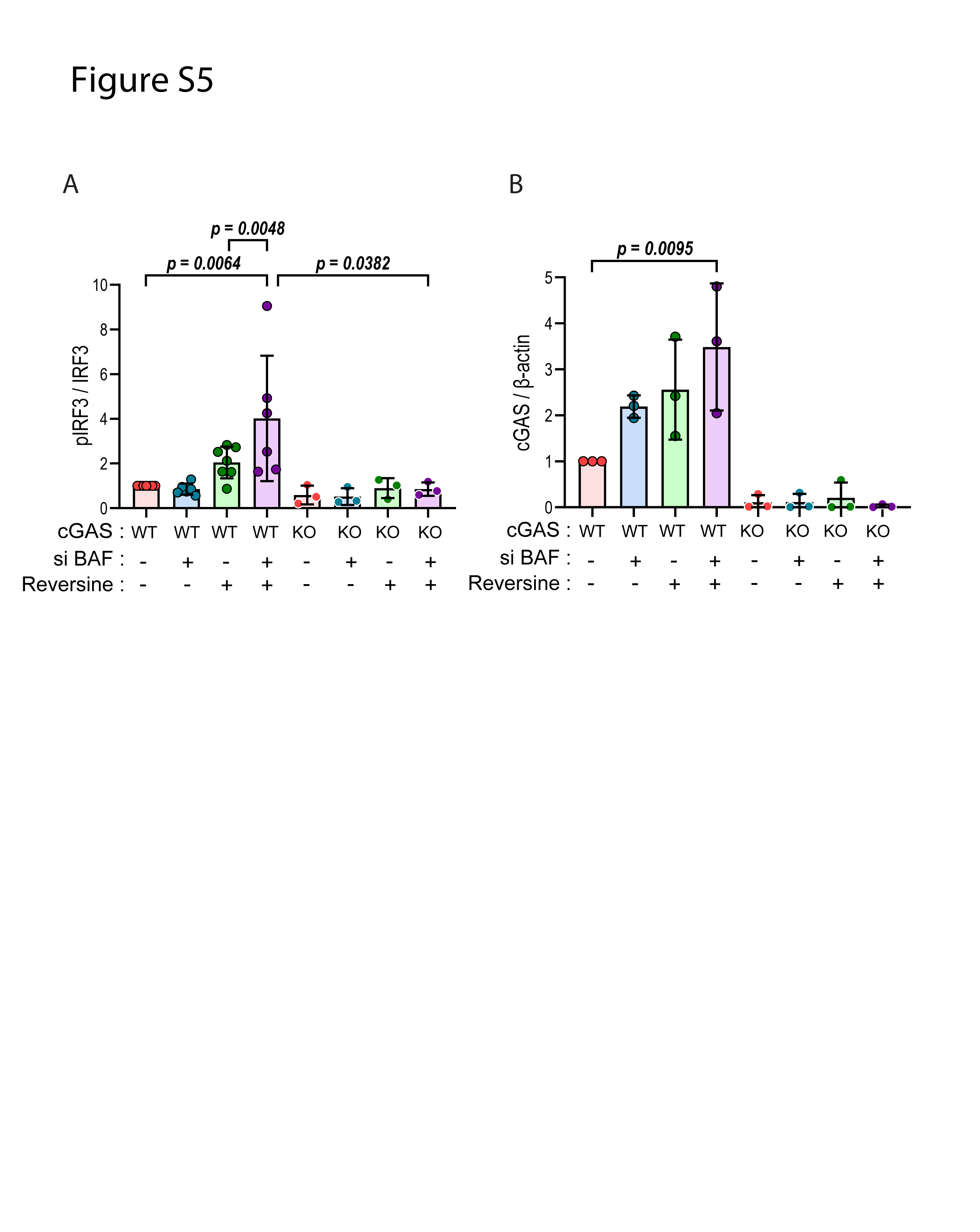

Supplement: S5 Fig — A. Ratios of pIRF3/ IRF3 signals. Averages of at least 3 experiments (6 or 7 for cGAS WT cells) ±SD are shown. B. Ratios of cGAS/ β-actin signals. Averages of 3 experiments ±SD are shown. p-values are from 2-way ANOVA. (TIF) [file pgen.1012191.s005.tif]

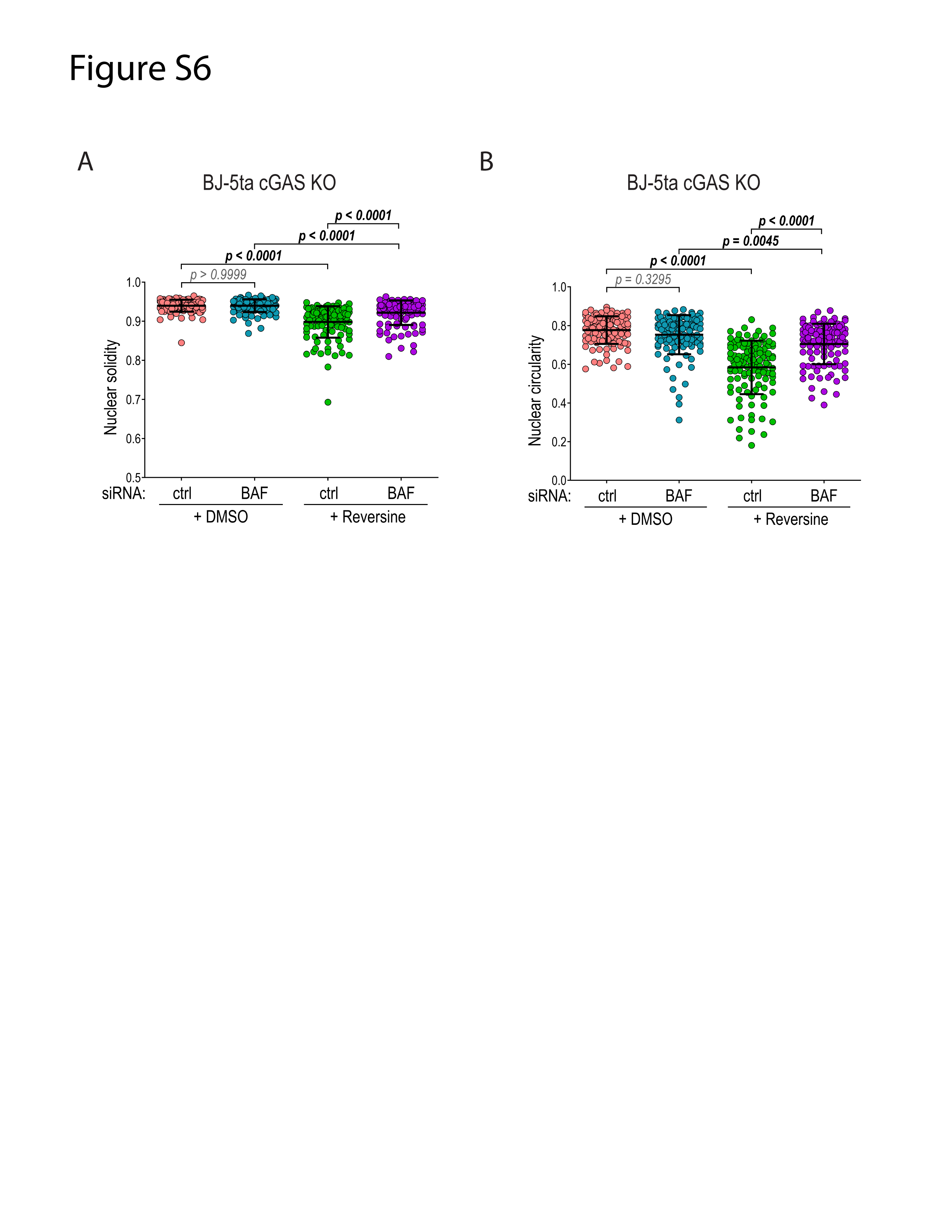

Supplement: S6 Fig — Nuclear solidity (A) and nuclear circularity (B) were measured from cells treated as indicated and processed by immunofluorescence as in Fig 2. Between 111 and 115 cells per condition were analyzed. Averages ±SD and p-values from one-way ANOVA are shown. (TIF) [file pgen.1012191.s006.tif]

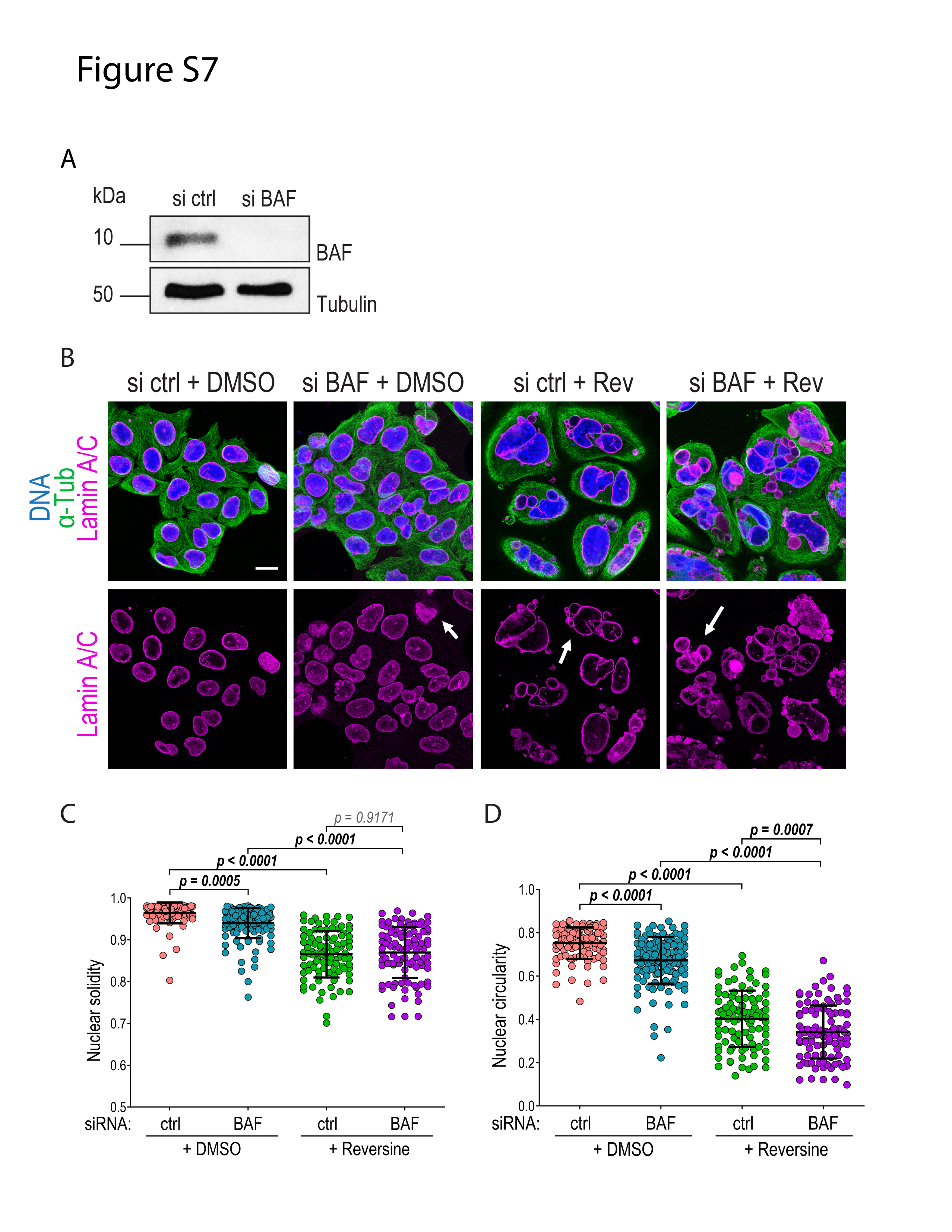

Supplement: S7 Fig — A. Western blotting showing the depletion of BAF 4 days after transfection with siRNA. B. HeLa cells were analyzed by immunofluorescence to reveal structural nuclear defect (arrows). Scale bars: 20 μm. C-D. Nuclear solidity (C) and nuclear circularity (D) were measured from HeLa cells expressing GFP-cGAS and treated as indicated from images as in B. Between 96 and 131 cells per condition were analyzed. Averages ±SD and p-values from one-way ANOVA are shown. (TIF) [file pgen.1012191.s007.tif]

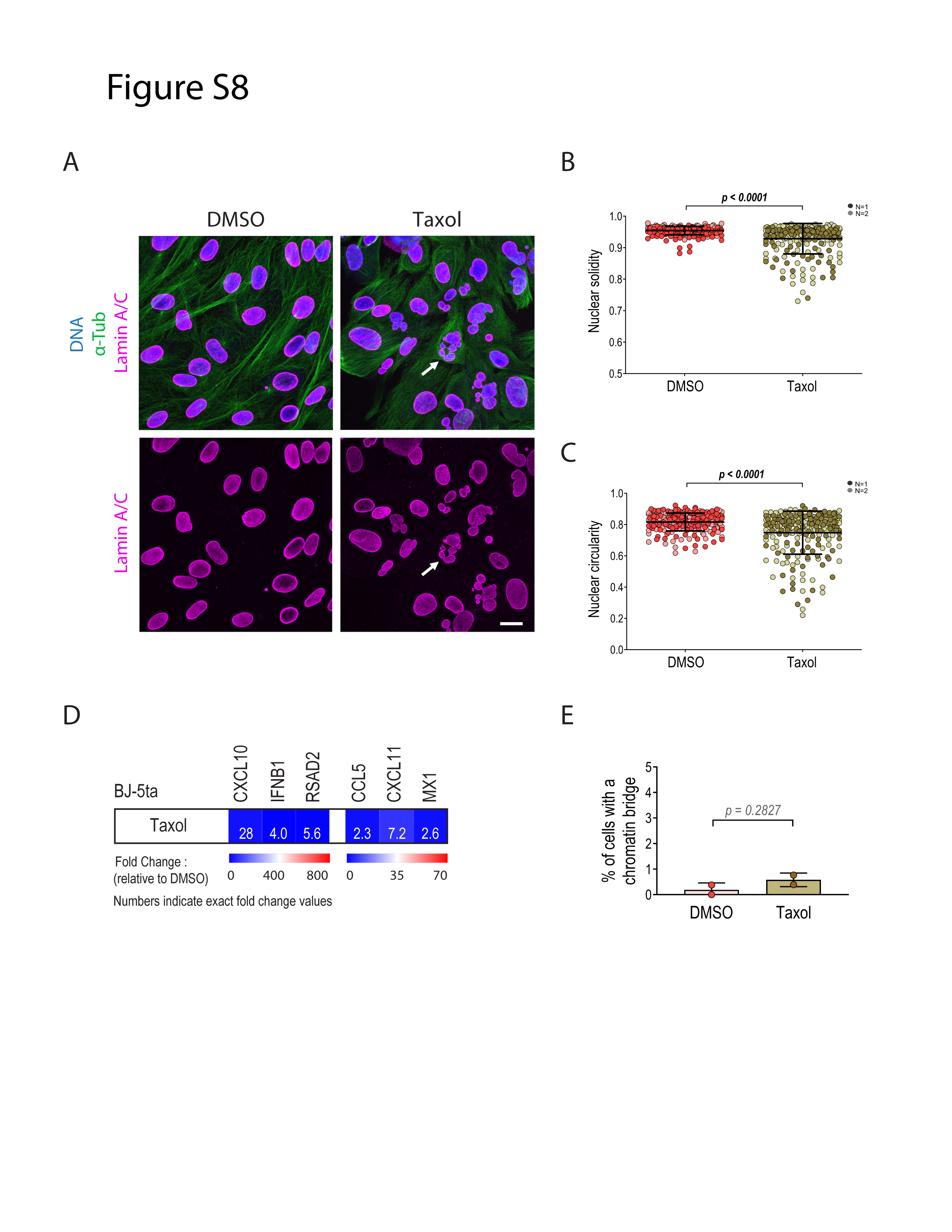

Supplement: S8 Fig — A. Immunofluorescence on cells treated as indicated to reveal nuclear defects (arrows). Taxol was used at 30 nM. Scale bars: 20 μm. B-C. Nuclear solidity (B) and nuclear circularity (C) were measured in cells treated as indicated. Averages ±SD from 2 independent experiments for which value sets (2 colors) were pooled on a same graph. More than 100 cells per condition per experiment were analyzed (Student’s unpaired T test shown). D. The expression of cytokines and ISGs was quantified by RT-qPCR after the indicated treatments in BJ-5ta WT cells. For each factor measured, heatmap colors indicate the fold changes relative to the control (DMSO) according to the color scale underneath. Averages from 2 independent experiments. Note that the nuclear defects following Taxol treatment are quantitatively similar to those obtained after Reversine treatment (Fig 2) but that the transcriptional response is much weaker (Fig 1). All cells were transfected with siRNA ctrl. E. Taxol treatment of BJ-5ta cells does not induce a marked increase in CBs. Averages from 2 independent experiments ±SD are shown. More than 500 cells per condition per experiment were analyzed (Student’s unpaired T test shown). (TIF) [file pgen.1012191.s008.tif]

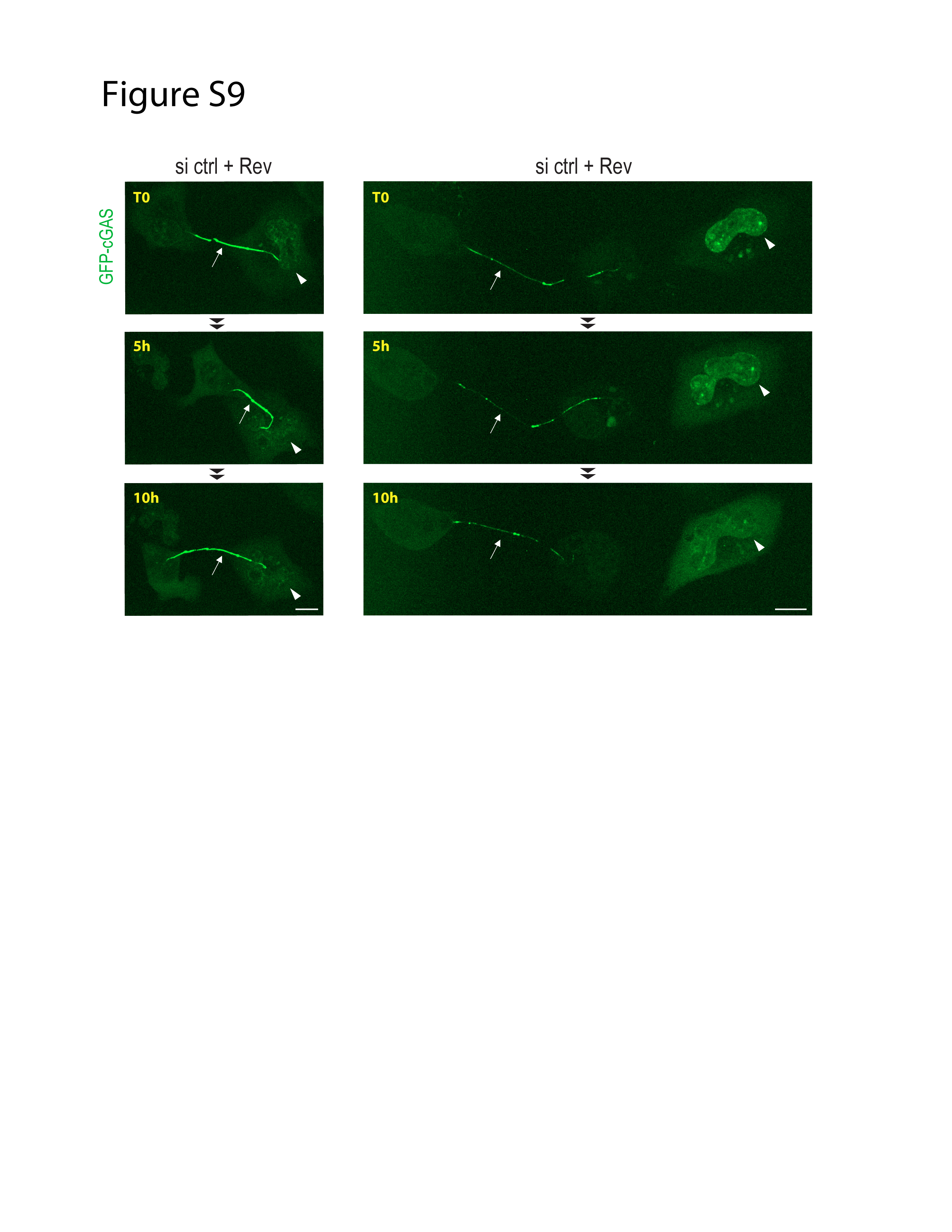

Supplement: S9 Fig — Two examples of time-lapse image series of MDA-MB-231 cells expressing GFP-cGAS and treated with Reversine are shown. GFP-cGAS remains strongly localized on post-mitotic CBs (arrows) for at least 10 hours while GFP-cGAS in the nucleus (arrowheads) decreases in intensity. Scale bars: 10 μm. (TIF) [file pgen.1012191.s009.tif]

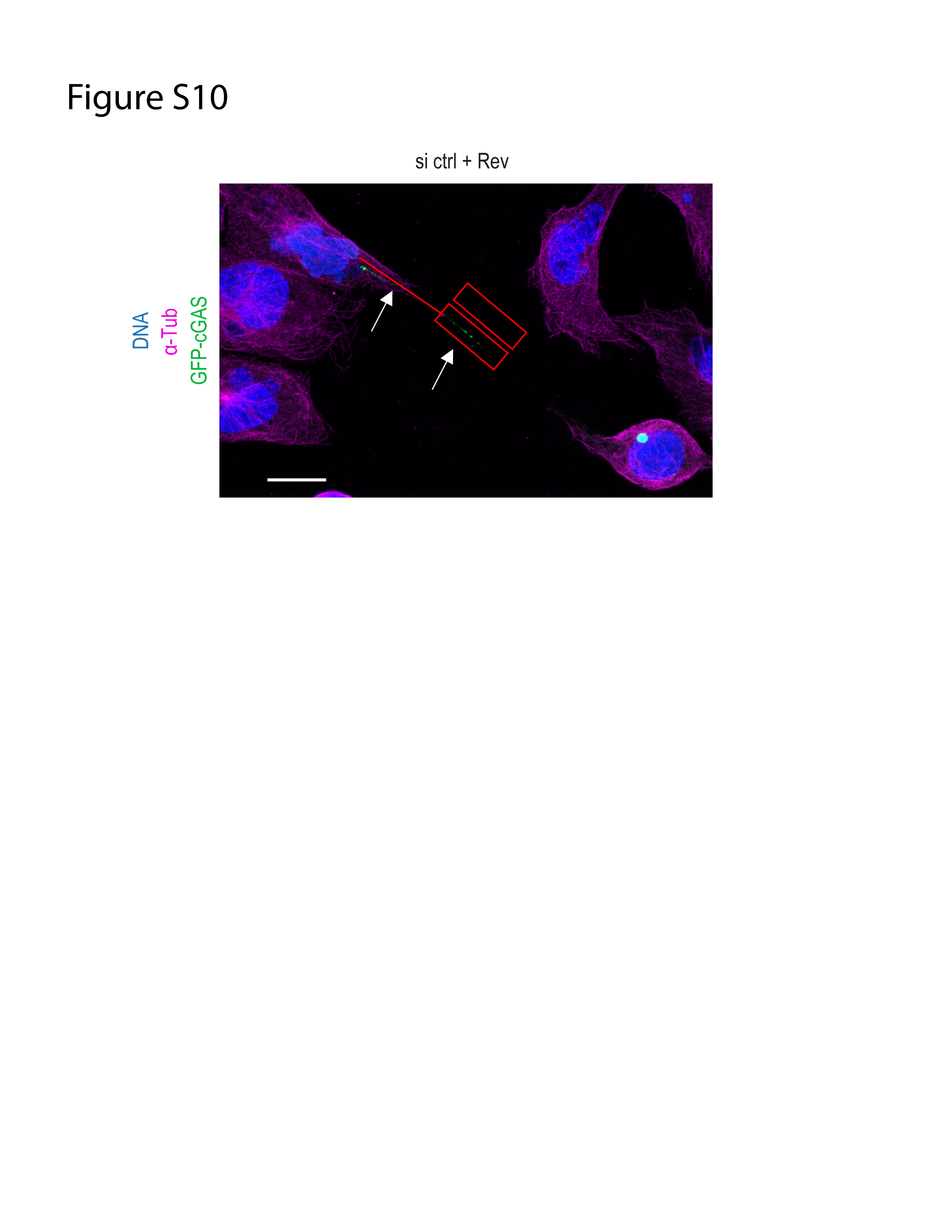

Supplement: S10 Fig — Immunofluorescence image from MDA-MB-231 cells expressing GFP-cGAS. A box of a fixed shape and area (red rectangle) was placed around the point of highest intensity of GFP-cGAS on the CB or bridge remnant. The mean fluorescence intensity of the GFP-cGAS signal within the box was measured and the mean intensity of the background measured from an immediately adjacent box was subtracted. The distance between the maximal GFP-cGAS intensity on CBs and the nearest nucleus to which it is connected was measured as indicated by the red line. Scale bar: 20 μm. (TIF) [file pgen.1012191.s010.tif]

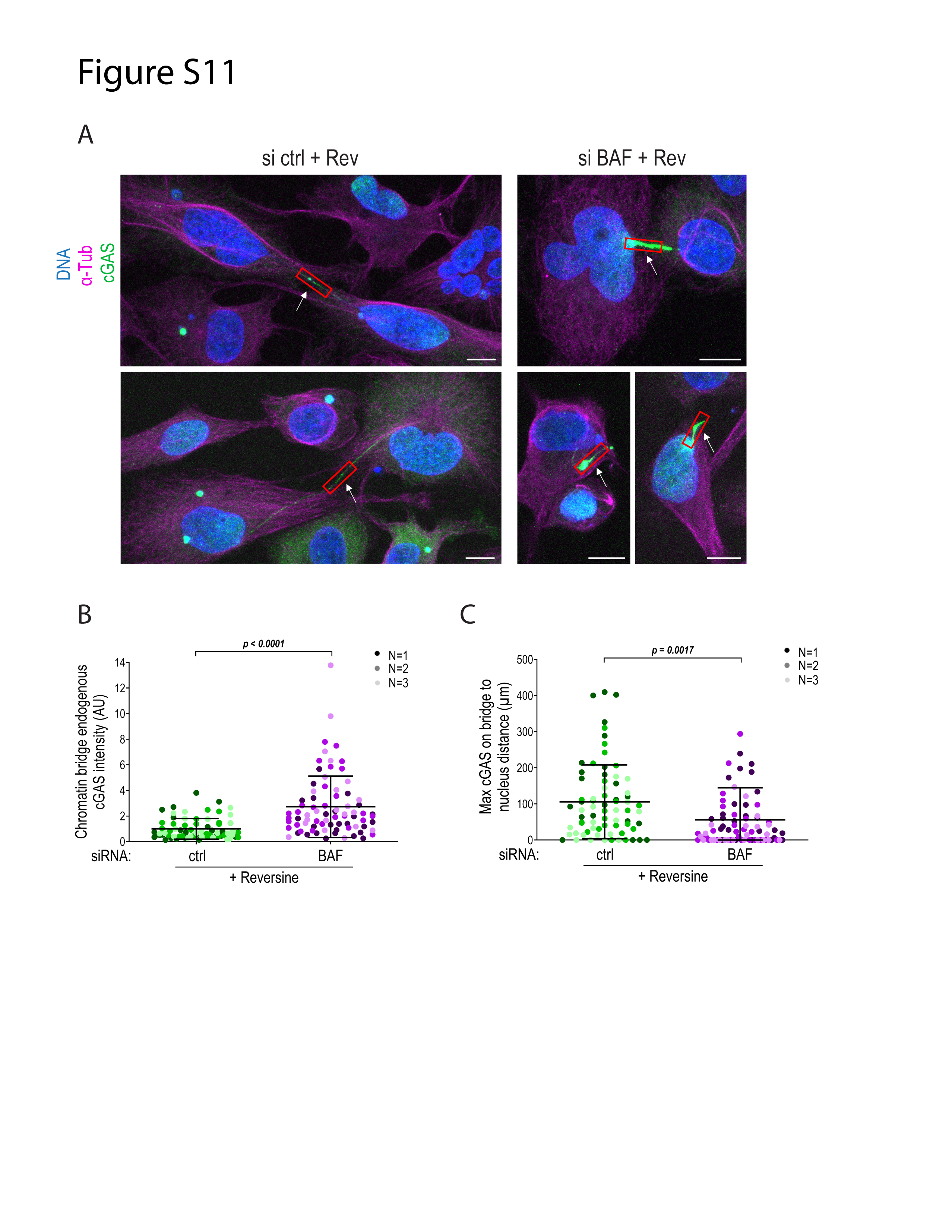

Supplement: S11 Fig — A. Examples of immunofluorescence images revealing endogenous cGAS on CBs or bridge remnants (red boxes). Scale bars: 10 μm. B. BAF depletion increases cGAS maximal intensity on CBs/bridge remnants in Reversine-treated cells. C. BAF depletion results in a shorter distance between maximal cGAS localization on CBs/bridge remnants and the nearest connected nucleus in Reversine-treated cells. For B-C, averages ±SD from 3 independent experiments are shown. The 3 value sets (3 colors) were pooled on a same graph. Between 23 and 28 bridges per condition per experiment were analyzed (Student’s unpaired T test shown). (TIF) [file pgen.1012191.s011.tif]

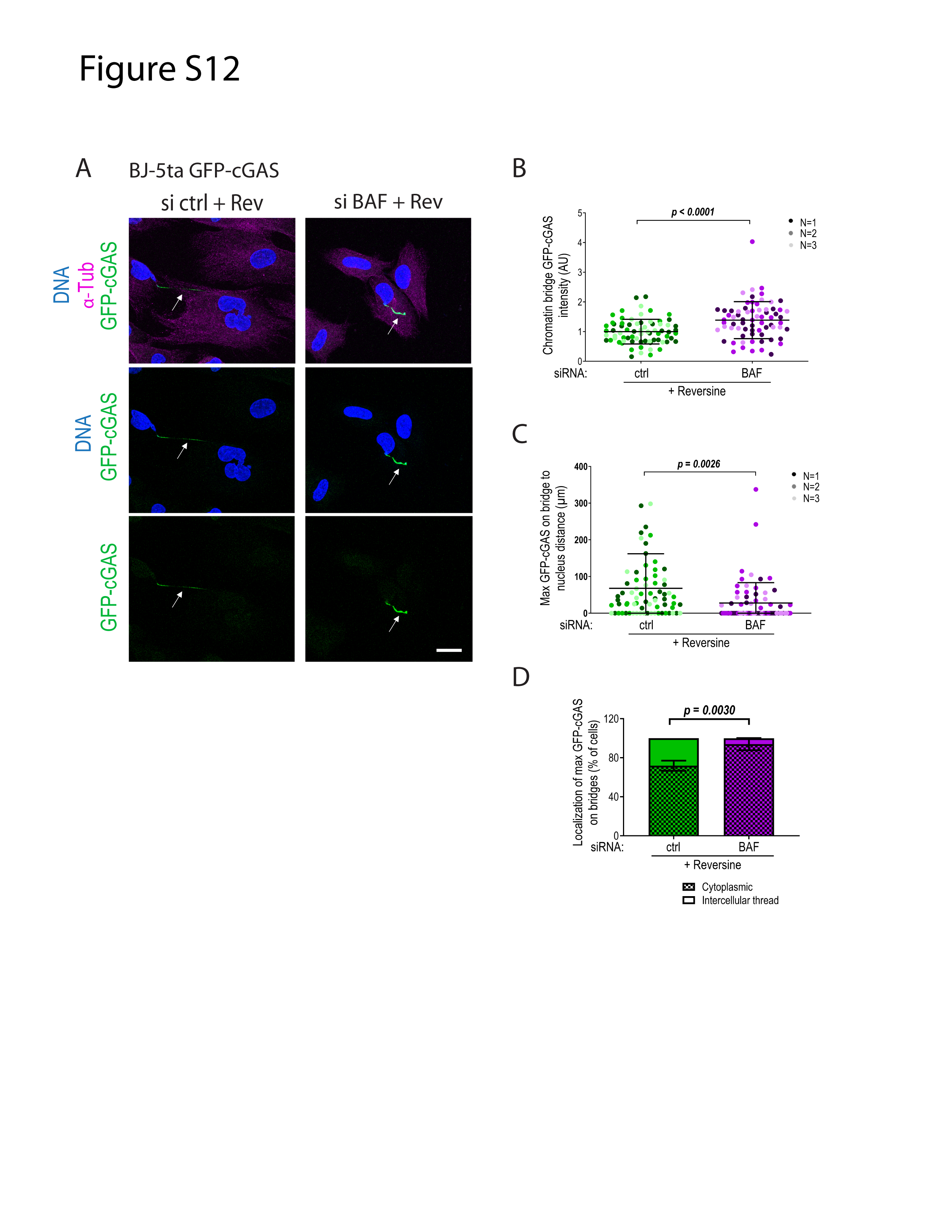

Supplement: S12 Fig — A. Examples of immunofluorescence images revealing GFP-cGAS recruitment on CBs or bridge remnants (arrows). Scale bar: 20 μm. B. BAF depletion increases GFP-cGAS maximal intensity on bridges of Reversine-treated cells. C. BAF depletion results in a shorter distance between maximal GFP-cGAS localization on CBs/bridge remnants and the nearest connected nucleus in Reversine-treated cells. D. BAF depletion increases the frequency of cytoplasmic maximal GFP-cGAS localization on CBs/bridge remnants upon Reversine treatment. Quantification of cytoplasmic vs non-cytoplasmic (intercellular thread) maximal GFP-cGAS localization on CBs/bridge remnants after the indicated treatments. For B-D, Averages ±SD from 3 independent experiments are shown. In B and C, the 3 value sets (3 colors) were pooled on a same graph. Between 18 and 28 bridges per condition per experiment were analyzed (Student’s unpaired T test shown). (TIF) [file pgen.1012191.s012.tif]

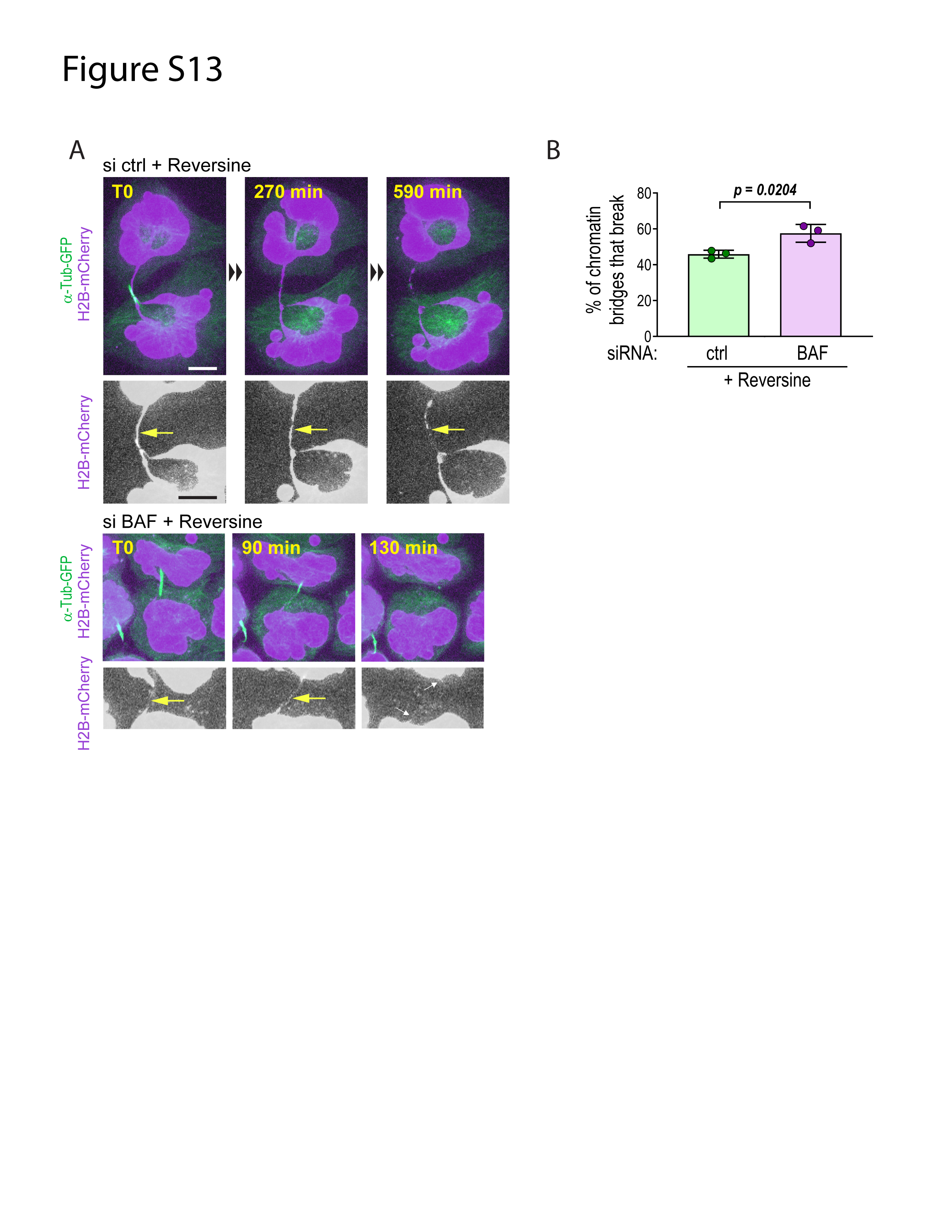

Supplement: S13 Fig — A. HeLa cells expressing H2B-mCherry and α-Tubulin-GFP were filmed after treatments as indicated. Examples of images from videos are shown. Yellow arrows indicate intact CBs and white arrows indicate bridge remnants. Scale bar: 10 μm. B. Quantification of the percentage of cells showing CBs that broke during the 12 h of filming. Averages ±SD from 3 independent experiments. Between 9 and 22 CBs per condition per experiment were analyzed (Student’s unpaired T test shown). (TIF) [file pgen.1012191.s013.tif]
