# Supplementary material for: Barrier-to-autointegration factor protects against the cGAS-STING response to chromatin bridges
Source: PLoS Genet. 2026 Jun 3;22(6):e1012191. doi: 10.1371/journal.pgen.1012191 (PMC13258145; doi:10.1371/journal.pgen.1012191)
Supplement: S1 Table — (DOCX) [file pgen.1012191.s018.docx]

**Supplementary Table 1. Primer oligonucleotides used for RT-qPCR analyses.**

| Gene | Forward | Reverse |
| --- | --- | --- |
| CXCL10 | gaaagcagttagcaaggaaaggt | gacatatactccatgtagggaagtga |
| CCL5 | tgcccacatcaaggagtattt | gggtgacaaagacgactgct |
| CCL20 | atgtgctgtaccaagagtttgc | tcaaagttgcttgctgcttc |
| CXCL11 | agtgtgaagggcatggcta | tcttttgaacatggggaagc |
| CXCL8 | agacagcagagcacacaagc | atggttccttccggtggt |
| IFIT2 | tggtggcagaagaggaagat | gtaggctgctctccaaggaa |
| IFIT1 | tccacaagacagaatagccagat | gctccagactatccttgacctg |
| IFNL1 | gggacctgaggcttctcc | ccaggaccttcagcgtca |
| IL6 | gatgagtacaaaagtcctgatcca | ctgcagccactggttctgt |
| TNF | cagcctcttctccttcctgat | gccagagggctgattagaga |
| RSAD2 | tgcttttgcttaaggaagctg | tctactttgcagaacctcacca |
| HPRT | tgatagatccattcctatgactgtaga | caagacattctttccagttaaagttg |
| ACTB | attggcaatgagcggttc | tgaaggtagtttcgtggatgc |
| BANF1 | tggctgggattggtgaag | tggccaaggacaacatagg |
| IFNB1 | cgacactgttcgtgttgtca | gaagcacaacaggagagcaa |
| IFI27 | gtggccaaagtggtcagg | ccaatcacaactgtagcaatcc |
| MX1 | ttcagcacctgatggccta | aaagggatgtggctggagat |
| ISG15 | gcgaactcatctttgccagt | ttcagctctgacaccgacat |
| IL1B | tacctgtcctgcgtgttgaa | tctttgggtaatttttgggatct |
| IFNA1 | cgaactctaccagcagctga | ccgcattcatcaggggagtt |
| CCL2 | agtctctgccgcccttct | gtgactggggcattgattg |
